# Interventions for Preventing Obesity in Children and Adolescents Aged 5–18 Years: An Overview of Nonrandomized Study Evidence Reported in 28 Systematic Reviews

**DOI:** 10.1111/obr.70090

**Published:** 2026-01-16

**Authors:** Francesca Spiga, Jelena Savović, Carolyn D. Summerbell, Hannah Picton, Theresa H. M. Moore, Deborah M. Caldwell, Julian P. T. Higgins

**Affiliations:** ^1^ Population Health Sciences, Bristol Medical School University of Bristol Bristol UK; ^2^ NIHR Applied Research Collaboration West (ARC West) at University Hospitals Bristol and Weston NHS Foundation Trust Bristol UK; ^3^ Department of Sport and Exercise Sciences Durham University Durham UK; ^4^ Fuse ‐ Centre for Translational Research in Public Health Newcastle upon Tyne UK

**Keywords:** BMI, children, nonrandomized study, obesity prevention

## Abstract

**Introduction:**

Community‐ and population‐level and policy interventions are commonly evaluated using nonrandomized studies (NRS), rather than randomized trials (RCTs). Recent Cochrane reviews of interventions for preventing childhood obesity have been restricted to RCTs, so less is known about the effectiveness of these more upstream interventions. To address this gap, we conducted an overview of reviews of NRS interventions (NRSI), which assessed change in BMI in children and adolescents aged 5–18 years and compared NRSI findings with those from RCTs.

**Methods:**

We searched five databases up to November 2024. Screening, data extraction, and quality assessment were performed using standardized tools.

**Results:**

We included 28 systematic reviews and identified 136 NRSI either based in school (*n* = 118), community (*n* = 4) or combined settings (*n* = 14) and evaluating policy (*n* = 48), education (*n* = 11), or a combined intervention (*n* = 77). Twenty‐six reviews included both NRSIs and RCTs; of these, 12 reported meta‐analyses. Findings were largely unchanged when we excluded the RCTs and re‐ran analyses. Overall, study‐level results from the NRSI favored the intervention group; a quarter favored the comparison group. The meta‐analysis summary effects from NRSIs were consistent with two recently published Cochrane meta‐analyses of RCTs of obesity prevention interventions.

**Conclusions:**

The results from this overview of reviews suggest researchers and policy makers can be confident in considering the results of robust nonrandomized study designs (evaluating their impact on BMI) alongside RCTs in their decision making. Although we identified a significant number of NRSIs for review, very few evaluations of upstream interventions were eligible for inclusion.

**Abbreviations**
BMI: body mass index
BMIp: body mass index percentile
CASP: Critical Appraisal Skills Programme
CI: confidence interval
CONSORT: Consolidated Standards of Reporting Trials
CRD: Centre for Reviews and Dissemination
EPOC: Effective Practice and Organization of Care
FI: fitness intervention
MD: mean difference
*N*
: number
n/a: not applicable
NR: nonreported
NICE: National Institute for Health and Care Excellence
NSLP: National School Lunch Programme
NRSI: nonrandomized studies of interventions
PA: physical activity
PICOS: Population, Intervention, Comparison, Outcome, and Study design
RCT: randomized controlled trials
RoB: risk of bias
ROBINS‐I: Risk of Bias in Nonrandomized Studies of Intervention
ROBIS: Risk of Bias in Systematic Reviews
SB: sedentary behavior
SD: standard deviation
SE: standard error
SMD: standardized mean difference
WHO: World Health Organization
zBMI: age‐ and sex‐standardized body mass index
τ: tau

## 1 Introduction

Globally, obesity is the fifth leading risk factor for mortality [1]. Prevalence rates of obesity among adolescents and children are increasing around the world, presenting a major public health problem [2]. Children living with overweight or obesity are at increased risk of living with overweight or obesity in adulthood [3, 4]. Childhood and adolescent overweight and obesity are risk factors for short‐ and long‐term morbidity, including type 2 diabetes, hypertension, coronary heart disease, depression, social isolation, and premature mortality [5].

A synthesis of data from 154 countries or regions found that one in five children or adolescents experienced excess weight and that rates of excess weight varied by regional income and Human Development Index [6]. Compared with 2000 to 2011, a 1.5‐fold increase in the prevalence of obesity was observed in 2012 to 2023. The rapid rise in overweight and obesity prevalence occurring in low‐ and middle‐income countries (LMICs), which are simultaneously undergoing a slower reduction in the prevalence of undernutrition, has fueled the double burden of malnutrition in LMICs [7]. In a separate analysis across high and middle‐income countries, the total costs of obesity ranged from 0.05% to 2.42% of the country's gross domestic product (GDP); consistent evidence indicated that the economic burden of obesity was substantial [8]. Policies to tackle population‐level obesity, including childhood obesity, have been introduced in many countries around the world. Most of these policies target upstream interventions and dietary intake behaviors (rather than physical activity behaviors). Examples include taxation policies on sugary beverages, which have been instituted in over 45 countries, cities, and regions across the world including in LMICs in the Americas, Africa, Middle East, Southeast Asia and Western Pacific [9]. Food policies in LMICs, mainly an increase in tariff rates on “unhealthy” foods (sugar and confectionery products as well as fats and oils) and governments' subsidies, are associated with a reduction in overweight and that these effects are more pronounced among poorer individuals [10]. Another example is the adoption of food labelling systems, which have been instituted and evaluated in at least 15 countries including LMICs (Brazil, Chile, Columbia, Mexico, and Uruguay) [5, 11, 12, 13, 14, 15].

Current evidence indicates that the contribution of social factors to health is as important as that of individual lifestyle factors [16]. Furthermore, community‐ and population‐based interventions are most effective at targeting the wider social determinants of health [17, 18, 19, 20, 21]. Research into effective interventions for preventing childhood obesity has included a wide range of study designs. Evidence to date from RCTs suggests that some school‐based interventions targeting dietary and physical activity behavior or lifestyle changes at the individual level may reduce obesity [22, 23]. The WHO Commission Ending Childhood obesity suggests that upstream interventions such as implementation of policies at community‐ and population‐level may be particularly important as they have actions at multiple levels of influence and may also be effective at preventing childhood obesity [5, 24]. While little evidence on the effectiveness of such upstream and policy interventions in terms of change in BMI is available from RCTs, a substantial amount of evidence is available from controlled studies with a nonrandomized design, which may provide insights into the effectiveness of such interventions. Although results from NRSIs often show larger intervention effects than those from RCTs, this is not always the case and there is a long‐standing debate about the use of the “hierarchy of evidence” in public health policy research [25, 26].

If good quality NRSIs in this field yield similar findings to RCTs, then added confidence can be placed on the results of systematic reviews of NRSIs, which are likely to remain the main evidence for upstream and policy interventions such as taxes and food labelling. Therefore, to complement the current evidence provided by systematic reviews of RCTs, we undertook an overview of systematic reviews of nonrandomized studies of interventions (NRSIs) to prevent obesity in children and extracted for re‐analysis a subset of comparative NRSIs that, except for the study design, would otherwise have been eligible for inclusion in two recent Cochrane reviews of RCTs [22, 23]. The overview sought to identify and understand the nonrandomized evidence base around whether population, community, and school‐based interventions are effective at preventing obesity in children through achieving reductions in body mass index (BMI) and to compare these effects with evidence from RCTs.

## 2 Methods

We undertook an overview of systematic reviews that included NRSIs evaluating the effect of interventions aimed at preventing obesity in children aged 5–18 years. This overview was conducted in line with the Cochrane Handbook of Systematic Reviews [27, 28]. The overview protocol was prospectively registered (PROSPERO: CRD42023420316).

### 2.1 Eligibility Criteria

We developed inclusion and exclusion criteria according to the Population, Intervention, Comparison, Outcome, and Study design (PICOS) framework.

#### 2.1.1 Participants/Population

Eligible systematic reviews addressed children and adolescents from the general population aged 5–18 years, in community or school settings, and focusing on children and adolescents only. We defined children and adolescents, participants aged 5–11 years and 12–18 years, respectively. If reviews included wider age groups, we required the effect of intervention on children's BMI to be reported separately.

#### 2.1.2 Intervention

We included any intervention aimed at preventing obesity, including dietary and/or activity interventions (i.e., increasing physical activity and reducing sedentary behavior) involving nutrition, education, lifestyle change, policy, social support, and combinations of these, implemented in any setting or via any medium. We excluded systematic reviews aimed at the treatment of obesity.

#### 2.1.3 Comparators Control

Eligible reviews included any active intervention or no intervention as comparators.

#### 2.1.4 Outcome

We included systematic reviews in which at least one BMI outcome was reported. Consistent with previous reviews of RCTs, our primary outcomes of interest were continuous or dichotomized measures of BMI and standardized BMI (zBMI), including BMI percentile (BMIp), reported in children and adolescents aged from 5 to 18 years.

#### 2.1.5 Study Design

We included systematic reviews reporting results of NRSI including nonrandomized experimental studies, interrupted time series (with or without control/counterfactual), controlled before and after studies, and other natural experiments but excluding modeling studies. Reviews that included both nonrandomized and randomized studies were eligible for inclusion. However, to allow comparison of results between NRSIs and RCTs, these reviews were included only if results from the nonrandomized studies were reported individually or separately.

For this overview, we defined systematic reviews as reviews with the following characteristics:

- A clear research question using the PICO format.
- Unambiguous, prespecified eligibility criteria (i.e., based on eligibility criteria being reported in a preregistered protocol or clearly stated in the review).
- A systematic literature search conducted in at least three databases.
- At least two reviewers contributing to study selection, data extraction, and risk of bias assessments (either independently or with one checking the work of the other).

### 2.2 Search and Selection of Systematic Reviews

To identify systematic reviews, we searched Medline, Embase and PsycInfo (via Ovid), the Cochrane Database of Systematic Reviews, and Epistemonikos, from inception to November 2024. We searched for terms relating to obesity, BMI, prevention, children, and NRSI. The search strategy was specific to each database (Table S1). All citations identified were uploaded to EndNote 20 (Clarivate) for duplicate removal and de‐duplicated results were then uploaded to Rayyan (rayyan.qcri.org). Two reviewers (F.S. and H.P.) independently selected eligible reviews. Any discrepancies were resolved by discussion.

### 2.3 Data Extraction and Risk of Bias Assessment for Included Reviews

A data extraction form was developed in Excel, which was first piloted on five reviews by two reviewers. As good agreement was achieved at the piloting stage, data extraction of studies characteristics was completed by a single reviewer, with uncertainties resolved by consulting a second reviewer. Numerical outcome data were extracted independently by two reviewers (F.S., H.P., J.S.) with discussion to resolve discrepancies. From each review, we extracted information on the participants and interventions (in the included studies) and other descriptive material. Risk of bias assessments of the included systematic reviews using the Risk of Bias in Systematic Reviews (ROBIS) tool [29] were performed by one reviewer (F.S., H.P. or J.S.) and checked by a second reviewer (F.S. or J.S.). The ROBIS tool addresses the following four domains: study eligibility criteria, identification and selection of studies, data collection and study appraisal, synthesis, and findings. Each domain consists of several questions, with five potential answers—“yes,” “probably yes,” “probably no,” “no,” or “no information.” The answers are combined to give each review an overall risk of bias rating of “low,” “high,” or “unclear” risk of bias.

### 2.4 Examination of the Included Primary Studies

In our protocol we planned to undertake synthesis at the level of the systematic review or meta‐analysis. However, to mitigate the potential for aggregation bias and double counting of studies analyzed in multiple reviews, we decided to examine the individual primary studies included in the systematic reviews. In this way, the included reviews can be viewed as the “source” for identification of eligible NRSIs. For this exercise, we defined “eligible NRSIs” more precisely as controlled nonrandomized studies of interventions (including interrupted‐time series) aiming to prevent obesity in children and young people aged between 5 and 18 years that reported at least one BMI outcome.

For each eligible NRSI included in an included systematic review, one reviewer extracted data on the participants' age, setting, and type of intervention as well as numerical results relating to the BMI outcomes, with uncertainties discussed with the senior author. For individual studies included in more than one systematic review, in case of inconsistency between reviews, we retrieved the study full text and extracted the data from the primary article. Where key characteristics or outcome results from an individual study were unclear or not reported, we also examined the original report for clarification.

For each eligible NRSI, the intervention setting was classified as “school,” “community,” or combined “school and community.” Community settings included population‐level settings. Studies in which the intervention had a component set in the home were categorized according to the primary setting. Interventions were categorized as “diet,” “physical activity,” and combined “diet and physical activity,” according to whether they were aimed at changing both diet and physical activity (i.e., increasing physical activity and/or decreasing sedentary behavior) or both, respectively. For each study, we categorized the mechanisms of change implemented by each intervention based on the socio‐economic model of health behaviors [30] as follows:

- **Educational** (i.e., interventions likely to change behavior of individuals). For example, where the studies are of educational interventions for children (and or the parent carers).
- **Policy** (i.e., interventions to change the environment or policy changes). For example, studies focusing on changes to school policy, environment, or regional/national policy changes affecting diet and activity.
- **Educational and policy**: studies in which there is a mix of individual and environmental interventions.

### 2.5 Strategy for Data Synthesis

We first summarized the characteristics of the included systematic reviews in tabular form. Subsequent syntheses were primarily based on eligible NRSIs included in the systematic reviews.

We undertook two types of quantitative syntheses based on the individual eligible NRSIs: a “within‐reviews” approach and an “across‐reviews” approach. The within‐reviews approach sought to provide estimates of intervention effect from the NRSIs included in each review (where possible). As part of this, we also compared the results from NRSIs with the results from NRSIs and RCTs combined. Specifically, from each review, we extracted the results of the meta‐analysis of NRSIs alone and, when available, also the meta‐analyses across NRSIs and RCTs, as reported by the reviews’ authors. If these results were not available, from each systematic review, we identified NRSIs and RCTs that reported data in a format that was suitable for inclusion in a meta‐analysis; for example, BMI or age‐ and sex‐standardized BMI (zBMI) mean difference (MD) or combined BMI/zBMI reported as standardized mean difference (SMD), with a measure of variance (standard error [SE], standard deviation [SD], or 95% confidence interval [CI]). We performed random‐effects meta‐analyses of results from NRSIs only and of results from NRSIs and RCTs combined where these were available. Note that none of the reviews reporting meta‐analyses included only NRSIs.

The across‐reviews approach began with a simple yet comprehensive synthesis using vote counting by direction of effect (regardless of statistical significance) as recommended in the Cochrane Handbook for Systematic Reviews of Interventions [31]. For each study, we determined the direction of effect for each reported outcome. We present the vote counting syntheses overall and subgrouped by (1) type of intervention: diet, physical activity, or combined diet and physical activity; (2) by setting: school or community or both; and (3) by mechanisms of change (educational, policy, or both). We then sought to provide estimates of effect from the NRSIs and compared them with the results of the previous Cochrane reviews of RCTs [22, 23]. These analyses combined results of eligible NRSI (i.e., for which suitable data for meta‐analysis were available from systematic reviews), irrespective of which systematic review they had been included in. Meta‐analyses were conducted by age group (5–11 years and 12–18 years) and by type of intervention (diet, physical activity, combined diet, and physical activity). Studies including both age groups (i.e., 5–18 years age group) were included in both age groups meta‐analyses. For primary studies included in more than one systematic review and reporting results for the same BMI outcome(s), selection of results to be included in the meta‐analysis was based on the following criteria in the following order: (1) study reporting a complete versus incomplete effect estimate (e.g., MD with SE, SD, or CI versus MD with *p* value); (2) effect estimate reported for the whole group versus by subgroup; (3) larger versus smaller sample size; and (4) earlier publication date.

## 3 Results

### 3.1 Systematic Reviews Selection

**FIGURE 1:**
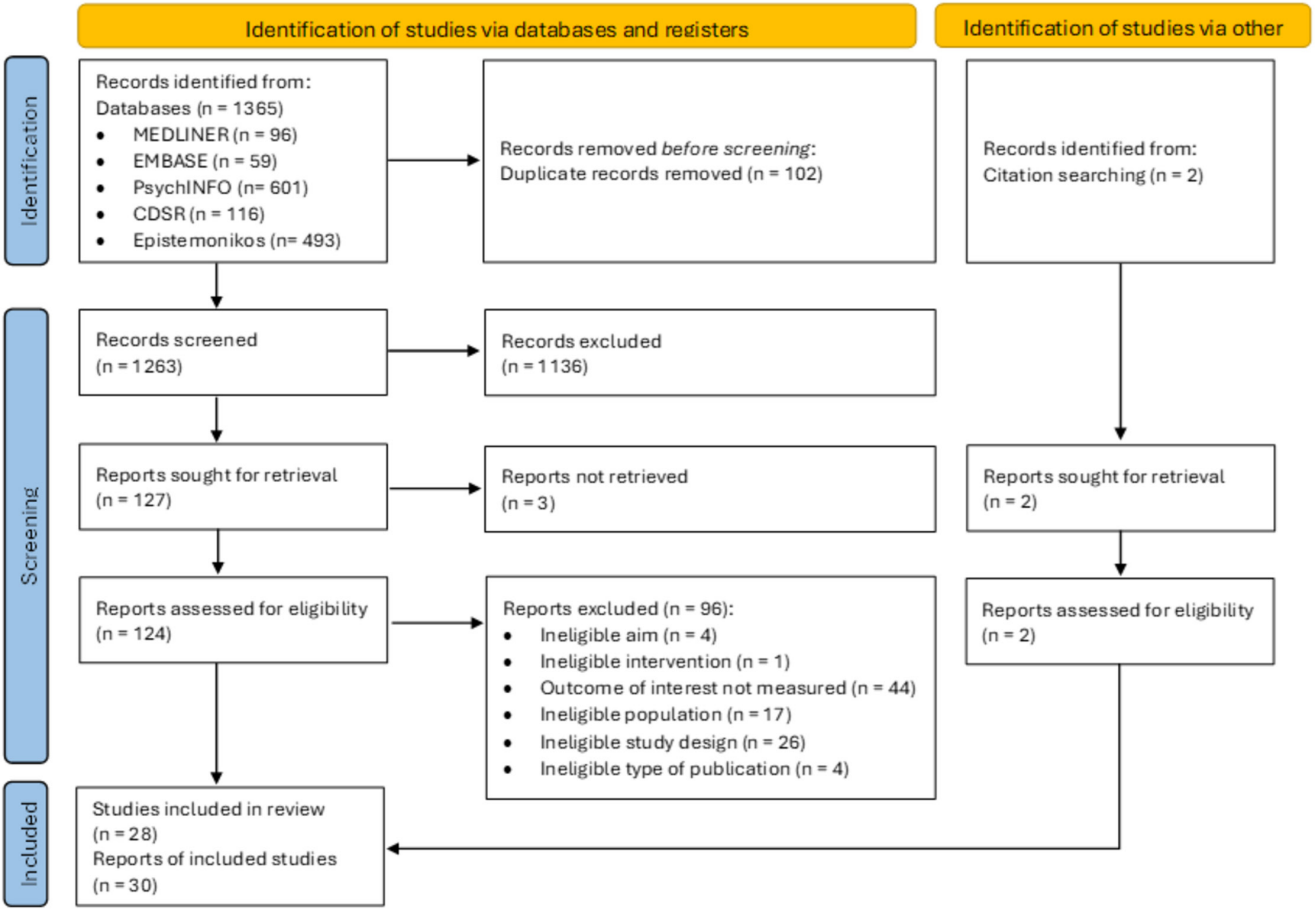
PRISMA flowchart.

The electronic searches yielded 1143 results (see PRISMA flowchart in Figure 1) and two records were identified through citation searching. After duplicates were removed, titles and abstracts of the remaining 1058 results were screened. We retrieved and screened the full text of 127 reviews, of which 97 reviews were excluded, and 28 reviews (31 records) met the eligibility criteria and were included in this overview. Full references of the 97 systematic reviews excluded at full‐text stage, along with reasons for their exclusion, are listed in Table S2.

### 3.2 Characteristics of the Included Systematic Reviews

Key characteristics of the 28 included systematic reviews are summarized in Table 1 and further details are provided in Table S3. The reviews were published between 2001 and 2024 and included between 6 and 146 studies (a grand total of 1001 studies, including studies reported in more than 1 review). Most of the reviews (26, 92.6%) included both RCTs and NRSI (e.g., difference‐in‐differences, interrupted‐time‐series, instrumental variables). One review only included NRSI, and one only included natural experiments.

**TABLE 1:** Characteristics of the included systematic reviews.

Study ID	Population of interest	Intervention(s) of interest	Review level type of interventions	Review level setting	Study design of the included studies^a^	Number of eligible NRSIs (total number of the included studies)
Atanasova 2022	Adults and children of any sex, ethnicity, socio‐economic status, and country of origin	Interventions targeting the built food environment (i.e., consumer and neighborhood food environments). The eligible study examined whether availability and distance to healthy food outlets influences residents' BMI	Diet	Any setting	Randomized control trials or quasi‐experimental studies (difference‐in‐differences, interrupted‐time‐series, instrumental variables)	1 (58)
Azevedo 2016	Children aged 0–17 years old from the general population	Sedentary behavior intervention, judged to target activities undertaken while sitting (e.g., TV viewing, video gaming). Studies were also included if they targeted other behaviors such as physical activity.	Physical activity	Any setting	Randomized or nonrandomized controlled trials	5 (67)
Balderas‐Arteaga 2024	Children attending from first to sixth grade of primary education	Interventions that integrated a class or lesson about nutrition and physical activity in the primary education curricula implemented for at least 1 year	Diet and/or physical activity	School‐based	Randomized controlled trials or quasi experimental studies design	3 (12)
Barnes 2018	Mothers and their 3‐ to 19‐year‐old daughters	Any community‐based intervention for mothers and daughters that targeted physical activity, fitness, nutrition, or adiposity	Diet and/or physical activity	Community‐based	Experimental trials, which assessed participants before and after exposure to an intervention, with or without a comparison group	1 (14)
Bramante 2019	The general pediatric population	Policies, programs, and built environment changes targeting a population	Diet and/or physical activity	Any setting	Natural experiments	6 (33)
Breslin 2023	School‐aged children, between the ages of 4 and 12 years	The Daily Mile intervention, involving children walking, running or wheeling outside for 15 min (approximately 1 mile) on a minimum of 3 days of the week	Physical activity	School‐based	Randomized controlled trials, quasi‐experimental studies, pilot studies, repeat measures, cross‐sectional, and natural experiments	3 (13)
Brown 2015	Children of South Asian ethnicity	Any type of lifestyle intervention (including diet and/or physical activity), of any length of follow‐up, that reported any anthropometric measure for children or adults of South Asian ethnicity, regardless of health status	Diet and/or physical activity	Any setting	RCTs, controlled clinical trials and before‐after studies	2 (29)
Campbell 2001	Children all aged less than 18 years at the commencement of the study	All types of intervention (dietary education and/or physical activity) other than drug or surgical interventions	Diet and/or physical activity	Any setting	Randomized‐controlled trials and nonrandomized trials with a concurrent control group	2 (7)
Errisuriz 2018	Children in primary (or elementary) school	Activity intervention implemented by a specialist physical activity teacher and designed to have an impact on children physical fitness, and/or body mass both in and out of class	Physical activity	School‐based	Experimental and quasi‐experimental studies	4 (12)
Feng 2017	Children and adolescent in primary and secondary schools	School‐based childhood obesity interventions at least 3 months based on primary and secondary schools in mainland China	Diet and/or physical activity	School‐based	Randomized and nonrandomized controlled trials	23 (76)
Godoy‐Cumillaf 2020	Children and adolescents (4–18 years of age) from Latin American countries	Physical activity interventions (physical endurance, sports, or alternative exercise such as games, dancing, optimized physical education classes; including or not diet intervention)	Physical activity (with or without a diet component)	Any setting	Randomized controlled trial, nonrandomized controlled trial, or single‐arm pre‐post study	2 (18)
Guerrero‐Magana 2024	Persons aged 6 years and over with an initial BMI greater than 18.5 kg/m^2^ (or equivalent in children and adolescents)	Interventions for the prevention of weight gain during festive and holiday periods. Interventions could include dietary advice, behavior change, implementation of exercise or physical activity, strategies to reduce sedentary behavior, self‐monitoring strategies (e.g., self‐weighing), supplements, intermittent fasting, or any other strategy that was focused on the prevention of weight gain.	Diet and/or physical activity	Any setting	Randomized controlled trials, cluster RCTs and nonrandomized controlled trials	3 (12)
Jacob 2021	Adolescents aged 10–19 from high‐income countries	Health educational intervention defined as “any combination of learning experiences designed to facilitate voluntary adaptations of behavior conducive to health”	Diet and/or physical activity	Any (educational) setting	Randomized controlled trials and nonrandomized controlled trials	9 (33)
Katz 2008	Children aged 3–18 in a school setting	Interventions aimed to prevent unnecessary weight gain or manage weight, related to nutrition, physical activity, reduction in television viewing or combinations of these	Diet and/or physical activity	School‐based	Randomized controlled trial, nonrandomized controlled trial, or single‐arm pre‐post study	3 (19)
Kornet Van der Aa 2017	Adolescents (mean age of ≥12 years, maximum age 18 years) from socio‐economically disadvantaged backgrounds	Obesity prevention and treatment programs for adolescents from socioeconomically disadvantaged backgrounds	Diet and/or physical activity	Any setting	Randomized or nonrandomized controlled trial or studies with a pre‐post design without control group	1 (14)
Mack 2017	Children between 7 and 15 years of age and of all weight categories	Every kind of video or computer game dealing with the topic of nutrition, physical activity, and obesity	Diet and/or physical activity	Any setting	Randomized and nonrandomized, qualitative and quantitative studies with and without comparison groups, pre‐post designs, and mere observational studies with any sample size	3 (64)
Pineda 2021	School aged children ≤19 years of age	Interventions that focus on the school food environment and that aim to shape accessibility, affordability, desirability, and convenience of food acquirement and consumption to prevent obesity/improve dietary intake	Diet (with or without a physical activity component)	School‐based	Randomized controlled trials and nonrandomized controlled trials	1 (100)
Podnar 2021	School‐age children from 6 to 12 years of age (mean age at the start of the study) from the general population	Interventions that aim to prevent obesity by increasing physical activity and/or physical fitness, or by reducing sedentary behavior, performed primarily in school setting, with follow‐up of at least 12 weeks from the start of the intervention	Physical activity (with or without a diet component)	School‐based	Randomized or nonrandomized control trial, controlled before and after study or natural experiment	68 (146)
Rochira 2020	Children attending school, aged 6–13 years	School gardening projects for primary school students	Diet and/or physical activity	School‐based	Randomized controlled trials, quasi‐experimental, and observational studies	3 (33)
Smit 2023	Children 6‐ to 12‐year‐olds at the start of the intervention	Primary school‐based obesity prevention interventions including interventions in multiple settings with a duration of ≥ 6 months and outcomes measured at least 1‐year follow‐up.	Diet and/or physical activity	School‐based	Randomized controlled trials and studies with other controlled experimental and observational designs	5 (19)
Spill 2024	School children, kindergarten to 12th grade	Community Eligibility Provision: universal free school meals (breakfast, lunch, or both)	Diet	School‐based	Nonrandomized intervention studies	1 (6)
Vega‐Salas 2023	Children in Latin America and the Caribbean	All interventions, including the introduction of policies, and/or regulations aiming at modifying obesity/overweight by changing food and/or built environment within and around the schools	Diet and/or physical activity	School‐based	Randomized and nonrandomized controlled trials and, cohort studies	5 (9)
von Philipsborn 2019	Any participants, including adults, adolescents and children, regardless of their weight, health status, and their country of residence	Any intervention intended to reduce or have potential effects on the consumption of SSB or sugar‐sweetened milk, or their adverse effects on health, implemented at the environmental level	Diet	Any setting	Randomized controlled trials, nonrandomized controlled trials, controlled before‐after, interrupted‐time‐series, repeated measures studies	2 (58)
Waters 2014	Children less than 18 years at the commencement of the study, including studies where children were part of a family group receiving the intervention	Interventions or programs that involved diet and nutrition, exercise, and physical activity including lifestyle and social support (educational, health promotion [i.e., “community‐based interventions”], psychological/family/behavioral therapy/counseling/management strategies)	Diet and/or physical activity	Any setting	Controlled trials (with or without randomization)	6 (55)
Williams 2013	Children aged 4–11 years participating in full time in education	National, regional, and school specific, diet‐, or physical activity‐related school policies either alone or as part of intervention programs	Diet and/or physical activity	School‐based	Randomized controlled trials, controlled before and after studies and interrupted time series, cohort and cross‐sectional studies	3 (21)
Wolfenden 2014	Participants from community samples of children and/or adults or specific population groups within a community defined on their demographics, ethnic, or socioeconomic characteristics.	Population‐based, whole of community interventions, which primarily seek to prevent population weight gain by targeting more than one determinant of population weight gain and included community consultation or engagement processes to inform intervention development or delivery (e.g., educational/health promotion/social marketing/management/organizational/counseling/policy or legislative reform strategies.	Diet and/or physical activity	Any setting	Randomized and nonrandomized designs with parallel control or comparison groups	6 (8)
Wolfenden 2017	Students in elementary, primary, secondary, middle, high, and central schools, where the age of students was typically between 5 and 18 years	Strategies with the intention of improving the implementation of health‐promoting policies, programs, or practices for physical activity, healthy eating, obesity prevention, tobacco use prevention or alcohol use prevention	Diet and/or physical activity	School‐based	Randomized and quasi‐randomized controlled trials, controlled before and after studies	1 (27)
Wolfenden 2022	Children aged 5–18	Strategies designed to improve the implementation of interventions (policies, practices, or programs) targeting student diet, physical activity, prevention of tobacco or alcohol use, or obesity	Diet and/or physical activity	School‐based	Randomized and quasi‐randomized controlled trials, controlled before and after studies	2 (38)

^a^ As reported by authors.

Abbreviations: BMI: body mass index; NRSI: nonrandomized study of intervention; RCT: randomized controlled trial.

We identified 136 unique eligible NRSIs (published in 141 reports) reporting at least one BMI outcome. The full reference list of eligible NRSIs is reported in Table S4. The number of eligible NRSIs included in each review ranged from one to 68, and those studies were published between 1986 and 2024. We found little overlap of eligible NRSIs between the reviews, with only six studies included in three systematic reviews, 26 studies included in two systematic reviews, and 104 studies included in only one systematic review (Table S5).

#### 3.2.1 Participants and Setting

Of the 28 included reviews, five included children, adolescents, and adults, and 23 only included children and/or adolescents. One review focused on children from South Asian ethnicity, one review included studies on mothers and daughters, one review included Latin American countries only, one review included Latin American and the Caribbean countries only, one focused on participants from high‐income countries only, and one review included only adolescents from socioeconomically disadvantaged backgrounds. The other 22 reviews included children and/or adolescents from the general population. Fifteen reviews (53.6%) only included studies set in school or other educational settings, 12 (42.9%) included studies in any setting, and one (3.6%) only included studies set in the community.

#### 3.2.2 Intervention Types

Four reviews (14.3%) included only studies of diet interventions (one of these with or without physical activity component); five (17.9%) included only studies of physical activity intervention (two of these with or without diet component); and 19 (67.9%) reviews included studies of diet, physical activity, or a combined diet and physical activity intervention.

#### 3.2.3 Methods of Risk of Bias Assessment of the Included NRSI

Of the 28 systematic reviews, 25 (89%) conducted a risk of bias assessment of the included NRSI using a formal risk of bias assessment tool (Table S3). Of these, eight reviews used the Risk of Bias in randomized trials (RoB1) [32] (or a modified version of it), five reviews used the quality assessment tool for quantitative studies of the Effective Public Health Practice Project [33] (or a modified version of it), five reviews used the Risk of Bias in Nonrandomized Studies of Interventions (ROBINS‐I) [34], two reviews used the Newcastle–Ottawa Scale for nonrandomized study designs [35] (or a modified version of it), one used the Critical Appraisal Skills Programme (CASP) Checklist for randomized controlled trials [36] and for cohort studies [36, 37], one used a nine‐item tool adapted from the Consolidated Standards of Reporting Trials (CONSORT) statement [38] and previously used quality criteria for methodology and reporting, one used a modified version of a quality assessment rubric based on Centre for Reviews and Dissemination (CRD) guidance [39], one used a modified version of the Downs and Black checklist [40], and one used a bespoken eight‐item scale tool.

#### 3.2.4 Risk of Bias Assessment of the Systematic Reviews

The overall risk of bias in each systematic review is reported in Table S3 and full assessment is reported in Table S6. Eight reviews (29%) were found to be at low risk of bias, 13 (46%) had unclear risk of bias, and seven (25%) were at high risk of bias. The main reasons for reviews being considered at high or unclear risk of bias were as follows: inappropriate restrictions in eligibility criteria based on study characteristics (five reviews), inadequate methods of literature searching and/or application of inappropriate restrictions during the study selection (14 reviews), inadequate data collection and risk of bias assessment of the included studies (eight reviews), and conducting inappropriate synthesis and/or not addressing studies' heterogeneity and/or bias in included studies (nine reviews).

### 3.3 Within‐Reviews Syntheses: Results of Meta‐Analyses of NRSIs and Comparison With Meta‐Analyses of Combined NRSIs and RCTs

**FIGURE 2:**
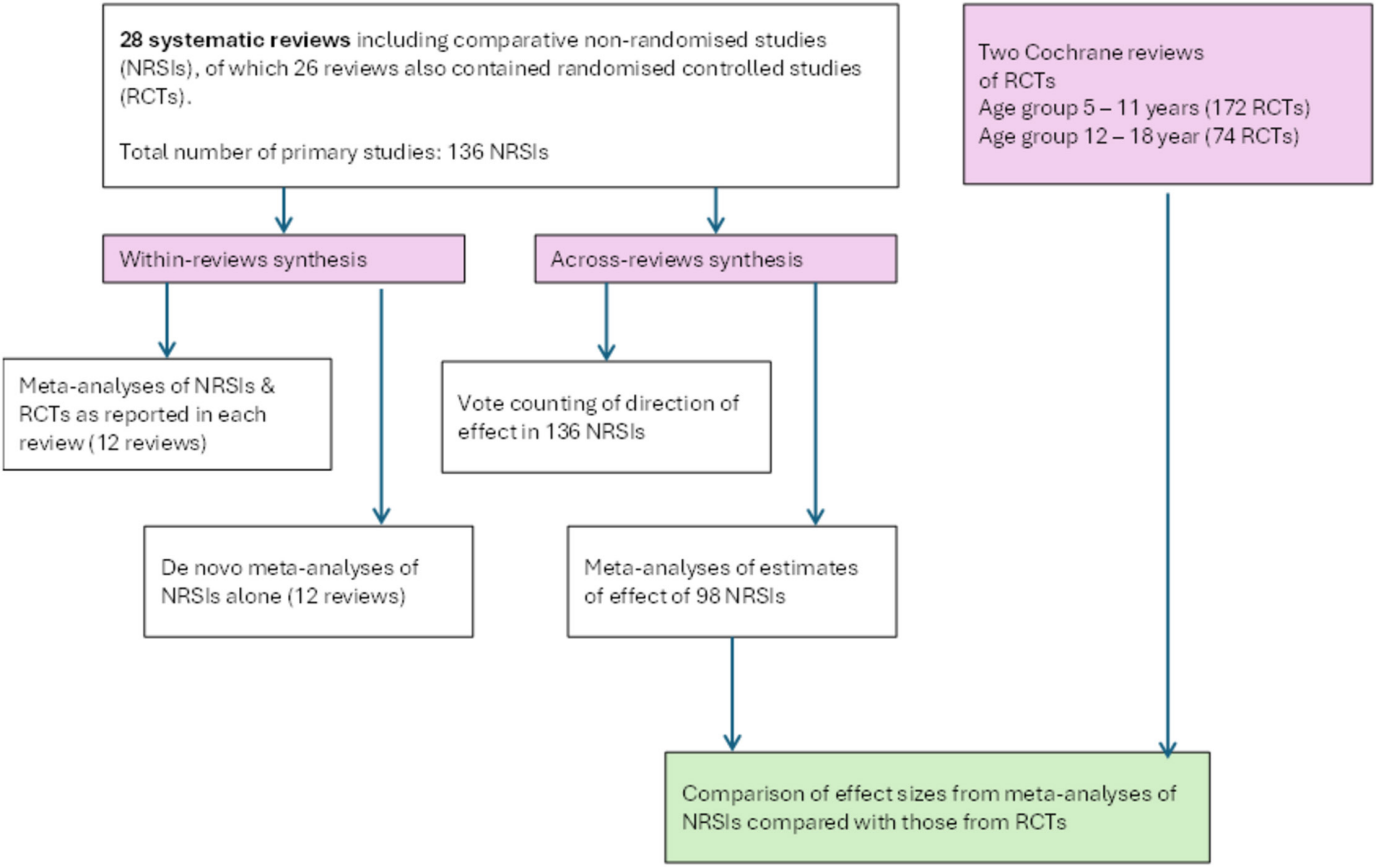
Diagram of within‐reviews and across reviews synthesis.

Twelve systematic reviews reported meta‐analysis results of NRSI alone or combined with RCTs (Table 2; Figure 2). In nine reviews, meta‐analyses of eligible NRSIs results showed a beneficial effect of the intervention on a BMI outcome; in seven reviews there was evidence of a beneficial effect of both the intervention and the control group, and in two reviews, meta‐analysis results showed both a beneficial or little to no effect of the interventions, depending on outcome(s) and subgroups.

**TABLE 2:** Syntheses within included reviews: comparison of results of NRSIs versus combined NRSIs and RCTs.

Review ID	Outcome	Meta‐analysis results from NRSIs	Meta‐analysis results from NRSIs & RCTs
Azevedo 2016	SMD	SMD (95% CI): 0.04 (−0.01, 0.15); 5 studies, 3260 participants^a^	SMD (95% CI): −0.06 (95% CI: −0.1, −0.02); 71 studies, 29,650 participants^b^
Balderas‐Arteaga 2024	zBMI	MD (95% CI): −0.05 (−0.09, −0.01); 1 study, 1109 participants	MD (95% CI): −0.14 (−0.25, −0.03), 4 studies, 4417 participants^b^
Brown 2015	SMD	SMD (95% CI): −0.32 (−1.89 to 1.24); 2 studies, 710 participants^a^	SMD (95% CI): −0.01 (−0.29, 0.28); 5 studies, 1038 participants^b^
Feng 2017	SMD	PA interventions: SMD (95% CI): 0.04 (−0.05, 0.13); 2 studies, 1759 participants^a^	PA interventions: SMD (95% CI): 0.05 (−0.04, 0.15); 4 studies, 1921 participants^b^
		DPA interventions: SMD (95% CI): −0.19 (−0.27 to −0.11); 3 studies, 4356 participants^c^	No RCTs
Gody‐Cumillaf 2020	SMD	SMD (95% CI): −0.09 (−0.18, −0.01); 2 studies, 2422 participants^a^	SMD (95% CI): −0.19 (−0.32, −0.07); 8 studies, 4864 participants^b^
Guerrero‐Magana 2024	zBMI	MD (95% CI): −0.05 (−0.11, 0.002); 2 studies, 255 participants^a^	MD (95% CI): −0.06 (−0.10, −0.01); 4 studies, 423 participants^b^
Jacob 2021	zBMI	MD (95% CI): −0.1 (−0.15, −0.05); 4 studies, 3072 participants^a^	MD (95% CI): −0.06 (−0.10, −0.03); 14 studies, 18,722 participants^b^
Katz 2008	BMI	MD (95% CI): −0.17 (−0.33 to −0.01); 3 studies, 3629 participants^a^	MD (95% CI): −0.29 (−0.45, −0.14); 8 studies, 10,752 participants^b^
Pineda 2021	zBMI	MD (95% CI): −0.20 (−0.26, −0.14); 1 study, n of participants is not reported	MD (95% CI): −0.12 (−0.15, −0.10); 5 studies, n of participants is not reported
Podnar 2021	SMD	FI + SB interventions: SMD (95% CI): −3.64 (−7.75, 0.48); 2 studies, 3410 participants^a^	FI + SB interventions: SMD (95% CI): −0.01 (−0.09, 0.07); 5 studies, n participants is unclear^b^
		PA interventions: SMD (95% CI): −0.31 (−0.55, −0.08); 18 studies, 10,544 participants^a^	PA interventions: SMD (95% CI): −0.04 (−0.09, 0.02); 38 studies, n participants is unclear^b^
		PA + SB interventions: SMD (95% CI): −0.18 (−0.39, 0.03); 7 studies, 7315 participants^a^	PA + SB interventions: SMD (95% CI): −0.07 (−0.13, −0.00); 24 studies, n participants is unclear^b^
		FI interventions: SMD (95% CI): −0.02 (−0.08, 0.03); 19 studies, 29,771 participants^a^	FI interventions: SMD (95% CI): −0.03 (−0.07, 0.00); 34 studies, n participants is unclear^b^
	zBMI	PA interventions: MD (95% CI): −0.13 (−0.17, −0.1); 14 studies, 10,039 participants^a^	PA interventions: MD (95% CI): −0.09 (−0.12, −0.06), 23 studies, n participants is unclear^b^
		FI interventions: MD (95% CI): −0.13 (−0.22, −0.04); 7 studies, 6252 participants^a^	FI interventions: MD (95% CI): −0.1 (−0.16, −0.03); 13 studies, n participants is unclear^b^
		PA + SB interventions: MD (95% CI): −0.04 (−0.14, 0.05); 4 studies, 17,634 participants^a^	PA + SB interventions: MD (95% CI): −0.05 (−0.09, −0.02); 17 studies, n participants is unclear^b^
Rochira 2020	BMI	MD (95% CI): −0.2 (−2.02, 1.62); 1 study, 104 participants	MD (95% CI): 0.13 (−0.94, 1.20); 2 studies, 188 participants^b^
	BMIp	MD (95% CI): −1.08 (−2.30, 0.15); 2 studies, 1989 participants^a^	MD (95% CI): −1.37 (−2.38, −0.37); 4 studies, 4593 participants^b^
	zBMI	MD (95% CI): −0.03 (−0.13, 0.07); 2 studies, 1276 participants^a^	MD (95% CI): −0.09 (−0.19, 0.01); 4 studies, 2062 participants^b^
Waters 2014	SMD	SMD (95% CI): −0.11 (−0.19, −0.03); 5 studies, 6001 participants^a^	MD (95% CI): −0.15 (−0.23, −0.08); 24 studies, 18,984 participants^b^

^a^ De novo meta‐analysis results including NRSIs only.

^b^ Meta‐analysis results including RCTs and NRSIs as reported in the systematic review.

^c^ Meta‐analysis results including NRSIs only as reported in the systematic review.

Abbreviations: BMI, body mass index; BMIp, BMI percentile; CI, confidence interval; FI, fitness intervention; MD, mean difference; NRSI, nonrandomized study of intervention; PA, physical activity; RCT, randomized controlled trial; SMD, BMI standardized mean difference; SB, sedentary behavior; zBMI, age‐ and sex‐standardized BMI.

Comparison of meta‐analysis results from eligible NRSIs with the results from NRSIs and RCTs shows that, of the 12 systematic reviews that reported meta‐analysis results, in nine reviews, results were concordant for one or more BMI outcomes or subgroups (six showing beneficial effect and three showing no effects); in two reviews, results were discordant (no effect in NRSIs and beneficial effect in NRSIs & RCTs); in two reviews, results were both concordant and discordant across the reported subgroup analysis.

### 3.4 Characteristics of the Included Primary Studies

Characteristics of the 136 eligible NRSIs (as extracted from the respective systematic reviews) are reported in Table S7.

#### 3.4.1 Participants and Setting

One hundred eight studies (79.4%) included children aged 5–11 years, 17 studies (12.5%) included adolescents aged 12–18 years and 11 studies (8.1%) included both children and adolescents aged 5–18 years. In the majority of the primary studies, interventions were delivered in schools (118 studies, 86.8%), in four studies (2.9%), the intervention was delivered at community level and in 14 studies (10.3%) both in schools and within the community. In 14 studies conducted in school and in two studies conducted in both school and the community, the interventions also had a component set within the home.

#### 3.4.2 Intervention Type and Mechanism of Change

Most of the primary studies (82, 60.3%) reported the effects of diet and physical activity interventions; 13 studies (9.6%) were on diet interventions only, and in 40 studies (29.4%), the intervention aimed at changing physical activity only. In one multiarm study (0.7%), the three intervention arms were a diet, a physical activity, and a combined diet and physical activity intervention. Eleven studies (8.1%) implemented educational interventions; in 48 studies (35.3%), the interventions were implementation of policy, and 77 studies (56.6%) implemented multicomponent interventions with both policy and educational interventions.

#### 3.4.3 Outcomes

From the 136 eligible NRSI included in the 28 systematic reviews, BMI outcomes reported were as follows: BMI (32 studies), zBMI (45 studies), BMIp (7 studies), BMI SMD (54 studies), proportion of participants who were living with obesity (%Ob, 16 studies), proportion of participants who were classified as overweight (%Ow, 8 studies), and proportion of participants who were classified as overweight or with obesity (%Ow/Ob, 12 studies).

#### 3.4.4 Risk of Bias and Quality Assessment of NRSIs

Risk of bias and quality assessment in NRSI is reported in Table S7. Of the 136 included studies, 103 (75.7%) were assessed for risk of bias, 25 (18.4%) were assessed for quality and 3 (2.2%) were assessed for both risk of bias and quality; no assessment was conducted on 5 studies (3.7%). Of the 103 studies that were assessed for risk of bias and/or quality, the majority (*n* = 75; 57%) were judged as high/severe risk of bias and/or low quality; two were judged as critical risk of bias (1.5%); 14 studies were of medium (or some concern) risk of bias; 19 (15.4%) were of low risk of bias and/or good or fair quality; among the studies included in more than one review, 18 (13.7%) were judged differently by different authors (mixed risk of bias/quality); judgment was unclear for three studies.

### 3.5 Across‐Reviews Syntheses: Findings of NRSIs by Broad Category of Participants and Interventional Approach

Across‐reviews synthesis included vote counting by direction of effect and comparison of estimates of effect from the NRSIs with the results of the previous Cochrane reviews of RCTs (Figure 2).

#### 3.5.1 Direction of Effects

We report summary results of direction of effect from individual studies by age group (5–11 and 12–18 years; Table 3). Then, for each age group, we report summary results by setting (school, community, school, and community), by type of intervention (diet, PA, and combined diet and PA) and by the mechanism of change implemented (educational interventions, policy interventions, and combined policy and educational interventions) (Supporting Information and Table S8). Of the 136 included primary studies, in 85 (62.5%), the direction of effect favored the intervention in all BMI outcomes, and in 31 (22.8%), the direction of effect favored the comparator. Ten studies (7.35%) reported mixed findings (favored both the intervention and the comparator): six studies by subgroups and four studies by outcomes. Direction of effect was not reported in 10 studies (7.35%) (Table 3).

**TABLE 3:** Syntheses across included reviews: direction of effects of interventions on BMI outcomes.

	Age group 5–11 years (119 studies)	Age group 12–18 years (28 studies)
Effect of the intervention	No. of studies (%)	No. of studies (%)
Direction: favors intervention	76 (63.9)	16 (57.1)
Direction: favors comparator	25 (21.0)	8 (28.6)
Direction: mixed across subgroups	5 (4.2)	1 (3.6)
Direction: mixed across outcomes	4 (3.4)	1 (3.6)
NR	8 (6.7)	2 (7.1)

Abbreviation: NR, not reported.

#### 3.5.2 Meta‐Analyses

We meta‐analyzed results from 98 (71%) of the eligible primary NRSI (i.e., only studies for which suitable data for meta‐analysis were available from systematic reviews), and we present those results alongside the results from RCTs previously reported elsewhere [22, 23].

##### 3.5.2.1 Age Group 5–11

In the age group 5–11 years (Table S9), results from NRSIs are consistent with those from RCTs, with very little to no effect of diet only interventions on BMI outcomes in both NRSIs and RCTs. We found little to no effect of activity only interventions in NRSIs and only some evidence of effect was observed in RCTs at medium‐term follow‐up (9 to < 15 months) in all BMI outcomes reported. In contrast, there was some evidence of beneficial effect of combined diet and physical activity interventions in NRSIs in all BMI outcomes reported, and some evidence of a beneficial effect in RCTs at short‐term (BMI; 12 weeks to < 9 months) and medium‐term (z‐BMI) follow‐up.

##### 3.5.2.2 Age Group 12–18

In the age group 12–18 years, none of the diet intervention studies reported results eligible for meta‐analysis (Table S10); results are inconsistent between physical activity alone and combined diet and physical activity interventions, with some evidence of effect in RCTs and little to no effect in NRSIs.

## 4 Discussion

### 4.1 Summary of Main Results

Our overview identified 28 systematic reviews of nonrandomized studies of interventions to prevent obesity in children and adolescents. Among these we evaluated the evidence from 136 NRSIs that reported BMI outcomes. Consistent with previous Cochrane reviews of RCTs with the same types of eligible intervention [22, 23], most of the NRSIs were conducted in children aged 5–11 years, most of the interventions were aimed at changing both diet and physical activity, and the majority were delivered in school. While we had hoped that, by looking at nonrandomized studies, we would identify a larger number of studies in nonschool settings, only a small proportion was set within the community or in both school and the community.

Our syntheses of direction of effect show that overall, more of the interventions were beneficial than not, with only a quarter favoring the comparison group. Subgroup analysis of direction of effect suggests that multicomponent interventions aimed at changing both diet and physical activity were most effective in reducing BMI gain in children aged 5–11 years and adolescents aged 12–18 years.

Meta‐analyses combining results of NRSIs and RCTs, reported in a subset of 12 included systematic reviews, did not produce results differing substantially from meta‐analyses that included only the NRSIs. Similarly, we found no substantial differences between meta‐analysis results from NRSIs and previous meta‐analyses of RCTs in Cochrane reviews [22, 23], reinforcing the results for both evidence bases. The proportion of studies in which the direction of the effect favored the intervention that were not included in the meta‐analysis (55.3%) is comparable to the number of those included in the meta‐analyses (65.3%), suggesting that our meta‐analysis results are not biased by selective reporting of positive results.

These results suggest that the results from NSRIs that met our inclusion criteria are very similar to those from RCTs. However, it remains the case that studies using random allocation (including cluster randomization) provide the most reliable evidence on comparative efficacy and, where possible, should be used in preference to other methods of allocation [41]. In most of the NSRI studies included in this review, it is unclear from the reporting why random allocation was not employed although we appreciate that reasons may include impracticability or lack of generalizability [42, 43]. We suggest that those responsible for collating and synthesizing the best available evidence for effectiveness of healthcare interventions should prioritize RCT designs where there is enough evidence and only include NSRIs and other study designs where there is a dearth of information from RCTs. This may be particularly true of upstream population‐level type interventions.

A limited number of high‐quality evaluations of upstream, policy‐level, population‐level interventions, which aimed to tackle childhood obesity were identified but did not meet the inclusion criteria for this review, mostly because of their study design and/or they did not assess change in BMI. These high‐quality evaluations used innovative study designs, for practical reasons, to substitute for a contemporaneous control group. Examples include the use of simulation modeling to estimate the potential effects on the prevalence of childhood obesity of (a) systematically offering preventive (and treatment) interventions to eligible children in England, based on weight or health status [44] and (b) a 20% and 30% ad valorem excise tax to sugar‐sweetened beverages in Brazil [45]. Another example includes the compilation of several macro‐ and micro‐level datasets (linked data sets) that provide information on policies or anthropometric data to estimate the potential effects of the policy, e.g., the effect of food policies, mainly tariff rates on “unhealthy” foods and governments' subsidies, in various countries [10].

### 4.2 Comparison With Previous Reviews on This Topic

Our updated findings are similar to earlier overviews of systematic reviews that evaluated the effectiveness of childhood obesity prevention interventions. Several overviews (combining evidence from both RCTs and NRSIs) observed either small or no effects in most obesity prevention interventions [46, 47, 48, 49]. Consistent with our findings, Flodgren et al. found that interventions targeting sugar‐sweetened beverage consumption and involving nutrition education could have beneficial effects on dietary behavior (but not on BMI) in adolescents [48].

Cauchi et al. found that interventions reducing sedentary behavior and increasing physical activity could impact on childhood BMI, which supports our findings that improving diet and increasing physical activity could improve childhood BMI [47]. Furthermore, Bahia et al. found that interventions aiming to reduce sedentary behavior, and including parental involvement, positively impact childhood BMI in the short‐term [46].

Two overviews found that multicomponent interventions were more effective than single‐component interventions. Cauchi et al. suggested that this was a result of maximizing component suitability and hence, maximizing the number of children receiving benefit (e.g., some children may not enjoy physical activity, but could fully engage with sedentary behavior components) [47]. This could explain the conflicting results for single‐component interventions in this overview. Bahia et al. argued that multicomponent interventions are probably only effective in the short‐term [46].

### 4.3 Strengths and Limitations

The greatest strength of this overview is the systematic approach through which it was conducted. The overview was conducted methodically and in line with guidance from the Cochrane Handbook for Systematic Reviews of Interventions. Searches were conducted across multiple databases, some of which specifically index systematic reviews. The search strategy was kept as broad as possible and returned most of the systematic reviews included in this overview.

Our overview has some limitations. First, as we sought only systematic reviews, we did not identify primary studies that had not been included in eligible systematic reviews. Second, when selecting systematic reviews, we designed inclusion and exclusion criteria to retrieve only systematic reviews that were rigorously conducted (e.g., data extraction and risk of bias assessment conducted by at least two reviewers). This restriction may have resulted in the exclusion of systematic reviews that included additional primary studies. Third, our results are constrained by the quality of reporting of the included systematic reviews. We relied upon details of the systematic reviews as reported in the main text and supplementary materials (when available), without contacting review authors for clarification. Similarly, for data from individual studies included in the identified systematic review, we relied upon details and results as reported by the systematic reviews, without consulting the individual study (unless specified) and without contacting the individual study authors for clarification. Fourth, our interest was in comparative nonrandomized studies, which are generally prone to residual confounding and other biases. Fifth, although differences across follow‐up times were a key finding in the recent Cochrane reviews, we did not conduct such subgroup analysis in this overview. Finally, many systematic reviews were excluded during the study selection process because they did not report BMI outcomes. Although BMI is the most accurate way to measure adiposity in children, excluding studies that used other anthropometric measures (e.g., weight gain, waist circumference) as well as systematic reviews reporting on other outcomes that are relevant to obesity prevention in children, such as changes in demand, purchases, consumption or other dietary/activity behaviors that are often measured in upstream and policy intervention studies, but not BMI outcomes, will have limited the number of population‐level studies that we might otherwise have included and influenced the findings of this overview.

## 5 Conclusions

The results from this overview of reviews suggest researchers can be confident in considering the results of robust nonrandomized study designs to evaluate interventions to tackle childhood obesity that include measuring change in BMI or another indicator of body fatness. Consistent with findings from RCTs, the multicomponent interventions from robust NRSIs we identified that aimed at changing both diet and physical activity interventions seem likely to be more effective at reducing child BMI than single‐component interventions (i.e., that only aimed to change diet or physical activity).

Although considerable evidence was collated in this overview of reviews, it is not possible to draw firm conclusions about all types of interventions that are effective at preventing childhood obesity. It is now widely recognized that a system‐based approach is needed to tackle obesity, with joined‐up policy initiatives across government departments [50, 51, 52]. This reflects the global guidance provided by the WHO [24]. However, very few evaluations of upstream, policy‐level, population‐level interventions to prevent childhood obesity (e.g., taxation on unhealthy foods and food labelling regulation) met the eligibility criteria for our review. Future efforts to examine evidence [42, 43]for such interventions would benefit from broader eligibility for NRSIs to allow for a more considered judgment on the totality of the evidence.

## Author Contributions

F.S., T.H.M.M., .J.S., D.M.C., and J.P.T.H. conceived the overall study. F.S., J.S., and H.P. screened references identified by the search, assessed studies for inclusion, and extracted data. F.S., J.S., and H.P. resolved conflicting review selections, controlled extracted data, independently appraised the methodological quality of the included reviews, and graded the credibility of evidence. F.S. and J.P.T.H. conducted the meta‐analyses and drafted methods and results. F.S. produced tables and figures. F.S. drafted the main manuscript and the summary. T.H.M.M., J.S., H.P., D.M.C., C.D.S., and J.P.T.H. read and commented on review drafts. All authors read and approved the final version.

## Funding

This work was supported by the NIHR (NIHR131572).

## Conflicts of Interest

The authors declare no conflicts of interest.

## Supporting information


**Data S1:** Supporting Information.

**Table S1:** Search strategies.
**Table S2:** Excluded systematic review records.
**Table S3:** Details of 28 included systematic reviews.
**Table S4:** Full references of the included individual studies (identified from the 28 systematic reviews).
**Table S5:** Overlap in primary studies (NSRI) across the 28 included systematic reviews.
**Table S6:** ROBIS assessment of the included systematic review.
**Table S7:** Characteristics of the 136 eligible primary studies (NRSIs) identified from the 28 systematic reviews.
**Table S8:** Syntheses across included reviews: direction of effects of interventions on BMI outcomes by setting, type of intervention and mechanism of change.
**Table S9:** Meta‐analysis results from RCTs and NRSIs in participants in the 5–11 years age group.
**Table S10:** Meta‐analysis results from RCTs and NRSIs in participants in the 12–18 years age group.

## Data Availability

The data that support the findings of this study are openly available in Figshare at https://figshare.com/, reference number 10.6084/m9.figshare.30637100.
