# Supplementary material for: Interventions for Preventing Obesity in Children and Adolescents Aged 5–18 Years: An Overview of Nonrandomized Study Evidence Reported in 28 Systematic Reviews
Source: Obes Rev. 2026 Jan 16;27(7):e70090. doi: 10.1111/obr.70090 (PMC13243340; doi:10.1111/obr.70090)
Supplement: Supplementary file 2 — Table S1: Search strategies. Table S2: Excluded systematic review records. Table S3: Details of 28 included systematic reviews. Table S4: Full references of the included individual studies (identified from the 28 systematic reviews). Table S5: Overlap in primary studies (NSRI) across the 28 included systematic reviews. Table S6: ROBIS assessment of the included systematic review. Table S7: Characteristics of the 136 eligible primary studies (NRSIs) identified from the 28 systematic reviews. Table S8: Syntheses across included reviews: direction of effects of interventions on BMI outcomes by setting, type of intervention and mechanism of change. Table S9: Meta‐analysis results from RCTs and NRSIs in participants in the 5–11 years age group. Table S10: Meta‐analysis results from RCTs and NRSIs in participants in the 12–18 years age group. [file OBR-27-e70090-s002.pdf]

Interventions for preventing obesity in children and adolescents aged 5-18 years: an overview of non-randomized study evidence reported in 28 systematic reviews

Francesca Spiga[1*], Jelena Savović[1,2], Carolyn D Summerbell[3,4], Hannah Picton[1], Theresa HM Moore[1,2] Deborah M Caldwell[1], Julian PT Higgins[1,2]

[1]Population Health Sciences, Bristol Medical School, University of Bristol, Bristol, UK

[2]NIHR Applied Research Collaboration West (ARC West) at University Hospitals Bristol and Weston NHS Foundation Trust, Bristol, UK

[3]Department of Sport and Exercise Sciences, Durham University, Durham, UK

[4]Fuse - Centre for Translational Research in Public Health, Newcastle upon Tyne, UK

***Correspondent author:**
Dr Francesca Spiga
Population Health Sciences, Bristol Medical School
University of Bristol
Canynge Hall
Whatley Road
Bristol
BS8 2PN
UK
Email: F.Spiga@bristol.ac.uk

Contents

Direction of effect sub-group analysis results 2
Subgrouping by age group 2
Subgrouping by setting 2
Subgrouping by type of intervention 3
Subgrouping by mechanism of change 3
Table S1. Search strategies 5
Table S2. Excluded systematic review records 8
Table S3. Details of 28 included systematic reviews. 18
Table S4. Full references of included individual studies (identified from the 28 systematic reviews). 29
Table S5. Overlap in primary studies (NSRI) across the 28 included systematic reviews. 39
Table S6. ROBIS assessment of included systematic review. 45
Table S7. Characteristics of the 136 eligible primary studies (NRSIs) identified from the 28 systematic reviews. 46
Table S8. Syntheses across included reviews: direction of effects of interventions on BMI outcomes by setting, type of intervention and mechanism of change. 59
Table S9. Meta analysis results from RCTs and NRSIs in participants in the 5-11 years age group. 61
Table S10. Meta analysis results from RCTs and NRSIs in participants in the 12-18 years age group. 64
References 66

Direction of effect sub-group analysis results

Subgrouping by age group

Of the 119 studies that included participants within the age-group 5-11 years (including 11 studies of participants in the 5-18 years age group), in 76 (63.9%) the direction of effect favoured the intervention and in 25 (21.0 %) favoured the comparator. Nine studies (7.6%) reported mixed findings (favoured both the intervention and the comparator): five studies by subgroups and four studies by outcomes. Direction of effect was not reported in eight studies (6.7%). Of the 28 studies that included participants within the age-group 12-18 years (including 11 studies of participants in the 5-18 years age group), in 16 (57.1%) the direction of effect favoured the intervention and in eight (28.6 %) favoured the comparator; two studies (7.2%) reported mixed findings: one by subgroups and one by outcomes. Direction of effect was not reported in two studies (7.1%).

Subgrouping by setting

Age group 5-11 years

Subgroup analysis by setting showed that among the 105 studies set in school, in the majority (67, 63.8%) the direction of effect favoured the intervention and in 21 (20.0%) the favoured the comparator. Nine studies (8.6%) reported mixed findings: five studies by subgroups and four studies

by outcomes. Direction of effect was not reported in eight studies (7.6%). Of the three studies set in the community, in one (33.3%) the direction of effect favoured the intervention, in one favoured the comparator and in one it was mixed across subgroups. Among the 11 studies set in both school and the community, in eight (72.7%) the direction of effect favoured the intervention and in three (27.3 %) favoured the comparator.

Age group 12-18 years

Subgroup analysis by setting showed that among the 22 studies set in school, in 14 (63.6%) the direction of effect favoured the intervention and in six (27.3%) favoured the comparator. One study (4.5%) reported mixed findings by outcomes and for; for one study the direction of the effect was not reported. Of the three studies set in the community, in one the direction of effect favoured the comparator and in one it was mixed across subgroups; for one study the direction of the effect was not reported. Among the three studies set in both school and the community, in two (66.7%) the direction of effect favoured the intervention and in one (33.3 %) favoured the comparator.

Subgrouping by type of intervention

Age group 5-11 years

Subgroup analysis by type of intervention showed that among the 12 studies implementing diet interventions, in four studies (33.3%) the direction of effect favoured the intervention, in four favoured the comparator, in two (16.7%) it was mixed across subgroups, and it was not reported in two studies. Of the 35 studies implementing a physical activity intervention, in 17 (48.6%) the direction of effect favoured the intervention, in 11 (31.4%) favoured the comparator, in three (8.6%) it was mixed across subgroups, and it was not reported in two studies. Of the 72 studies implementing combined diet and physical activity interventions, in 55 (76.4%) the direction of effect favoured the intervention, in ten (13.9%) favoured the comparator, in one (1.4%) it was mixed across subgroups and in four (5.6%) it was mixed across BMI outcomes; the direction of effect was not reported in two studies.

Age group 12-18 years

Subgroup analysis by type of intervention showed that among the four studies implementing diet interventions, in two studies (50%) the direction of effect favoured the intervention, in one favoured the comparator, and in one (25%) it was mixed across subgroups. Of the eight studies implementing a physical activity intervention, in four (50%) the direction of effect favoured the intervention, in two (25%) favoured the comparator, and in two it was not reported. Of the 16 studies implementing combined diet and physical activity interventions, in ten (62.5%) the direction of effect favoured the intervention, in five (31.3%) favoured the comparator, and in one (6.3%) it was mixed across BMI outcomes.

Subgrouping by mechanism of change

Age group 5-11 years

Subgroup analysis by mechanism of change showed that, among the 11 studies in which the intervention was educational, in six (54.5%) the direction of effect favoured the intervention, in three (27.3%) favoured the comparator, in one it was mixed across subgroups in one study (9.1%), and it was not reported in one study. Among the 43 studies in which the intervention involved implementing policy, in 22 (51.2%) the direction of effect favoured the intervention, in 12 (27.9%) favoured the comparator, in five (11.6%) it was mixed across subgroups and not reported in four studies. Among the 65 implementing a multicomponent intervention with both educational and policy implementation, in 48 (73.8%) the direction of effect favoured the intervention, in ten (15.4%)

favoured the comparator, it was mixed across outcomes in four studies (6.2%) and not reported in three studies.

Age group 12-18 years

Subgroup analysis by mechanism of change showed the in the one study in which the intervention was educational the direction of effect favoured the. Among the 12 studies in which the intervention involved implementing policy, in five (41.7%) the direction of effect favoured the intervention, in five favoured the comparator, in one (8.3%) it was mixed across subgroups and in one it was not reported. Among the 15 implementing a multicomponent intervention with both educational and policy implementation, in ten (66.7%) the direction of effect favoured the intervention, in three (15.4%) favoured the comparator, it was mixed across outcomes in one study (6.7%) and not reported in one study.

Table S1. Search strategies

**Ovid MEDLINE(R) Epub Ahead of Print and In-Process, In-Data-Review & Other Non-Indexed Citations and Daily November 11, 2024**
1. review.pt.
2. (medline or medlars or embase or pubmed or cochrane).tw,sh.
3. (scisearch or psychinfo or psycinfo).tw,sh.
4. (psychlit or psyclit).tw,sh.
5. cinahl.tw,sh.
6. ((hand adj2 search$) or (manual$ adj2 search$)).tw,sh.
7. (electronic database$ or bibliographic database$ or computeri?ed database$ or online database$).tw,sh.
8. (pooling or pooled or mantel haenszel).tw,sh.
9. (peto or dersimonian or der simonian or fixed effect).tw,sh.
10. (retraction of publication or retracted publication).pt.
11. or/2-10
12. 1 and 11
13. meta-analysis.pt.
14. meta-analysis.sh.
15. (meta-analys$ or meta analys$ or metaanalys$).tw,sh.
16. (systematic$ adj5 review$).tw,sh.
17. (systematic$ adj5 overview$).tw,sh.
18. (quantitativ$ adj5 review$).tw,sh.
19. (quantitativ$ adj5 overview$).tw,sh.
20. (quantitativ$ adj5 synthesis$).tw,sh.
21. (methodologic$ adj5 review$).tw,sh.
22. (methodologic$ adj5 overview$).tw,sh.
23. (integrative research review$ or research integration).tw.
24. or/13-23
25. 12 or 24
26. prevent*.af
27. exp Obesity/
28. obes*.af
29. (Overweight or over weight).af.
30. (BMI or body mass index).af.
31. Or/27-30 (changed from or/26-30)
32. 26 and 31
33. (child* or infant* or p?ediatr* or boys or girls or youth* or teen* or pre-teen* or young people or young person* or young adult* or adoles* or schoolage* or school age* or studen3* or pupil* or schoolchild* or school child*).af.
34. 32 and 33
35. 25 and 34
36. (Non-randomi#ed or qua#i-randomi#ed or qua#i-experiment or natural experiment or interrupted time series or controlled before-after or difference in difference*).af.
37. 35 and 36
Systematic review filter used – BMJ Best Practice (https://bestpractice.bmj.com/info/toolkit/learn-ebm/study-design-search-filters/)
**Ovid EMBASE November 11, 2024**
exp review/
(literature adj3 review$).ti,ab.

exp meta analysis/
exp "Systematic Review"/
or/1-4
(medline or medlars or embase or pubmed or cinahl or amed or psychlit or psyclit or psychinfo or psycinfo or scisearch or cochrane).ti,ab.
RETRACTED ARTICLE/
6 or 7
5 and 8
(systematic$ adj2 (review$ or overview)).ti,ab.
(meta?anal$ or meta anal$ or meta-anal$ or metaanal$ or metanal$).ti,ab.
or/9-11
prevent*.af.
exp obesity/
obes*.af.
(overweight or over weight).af.
(BMI or body mass index).af.
Or/14-17
13 and 18
(child* or infant* or p?ediatr* or boys or girls or youth* or teen* or pre-teen* or young people or young person* or young adult* or adoles* or schoolage* or school age* or student* or pupil* or schoolchild* or school child*).af.
19 and 20
9 and 21
(Non-randomi#ed or qua#i-randomi#ed or qua#i-experiment or natural experiment or interrupted time series or controlled before-after or difference in difference*).af.
22 and 23
Systematic review filter used – BMJ Best Practice (https://bestpractice.bmj.com/info/toolkit/learn-ebm/study-design-search-filters/)
**Ovid PsycINFO November 11, 2024**
prevent*.af.
exp obesity/
obes*.af.
(overweight or over weight).af.
(BMI or body mass index).af.
Or/2-5
1 and 6
(child* or infant* or p?ediatr* or boys or girls or youth* or teen* or pre-teen* or young people or young person* or young adult* or adoles* or schoolage* or school age* or student* or pupil* or schoolchild* or school child*).af.
7 and 8 (new)
(Non-randomi#ed or qua#i-randomi#ed or qua#i-experiment or natural experiment or interrupted time series or controlled before-after or difference in difference*).af.
9 and 10
(((comprehensive* or integrative or systematic*) adj3 (bibliographic* or review* or literature)) or (meta-analy* or metaanaly* or "research synthesis" or ((information or data) adj3 synthesis) or (data adj2 extract*))).ti,ab,id. or ((review adj5 (rationale or evidence)).ti,ab,id. and "Literature Review".mp.) or (cinahl or (cochrane adj3 trial*) or embase or medline or psyclit or pubmed or scopus or "sociological abstracts" or "web of science").ab. or ("systematic review" or "meta analysis").mp
11 and 12

Supporting information

Systematic review filter used – University of Texas<br>https://libguides.sph.uth.tmc.edu/search_filters/ovid_psycinfo_filters
**Epistemonikos**
Prevent* AND (obes*) OR (overweight) OR (over weight) OR (BMI) OR (body mass index) AND (((child) OR (infant*) OR (p?ediatr*) OR (boys) OR (girls) OR (youth*) OR (teen*) OR (pre-teen*) OR (young people) OR (young person*) OR (young adult*) OR (adoles*) OR (schoolage*) OR (school age*) OR (student*) OR (pupil*) OR (schoolchild*) OR (school child*))) AND (non-randomised) OR (non-randomized) OR (quazi-randomised) OR (quasi-randomised) OR (quazi-randomized) OR (quasi-randomised) OR (quasi-experiment) OR (quazi-experiment) OR (natural experiment) OR (interrupted time series) OR (controlled before-after) OR (difference in difference*)
**Cochrane Database of Systematic Reviews**
Mesh descriptor: [Obesity] explode all trees
Prevent*:ti
(obes* or overweight or over weight)
(BMI or “body mass index”)
#2 and (#1 or #3 or #4)
(child* or infant* or pediatr* or paediatr* or boys or girls or youth* or teen* or pre-teen* or "young people" or "young NEXT person" or "young NEXT adult" or adoles* or schoolage* or "school NEXT age" or student* or pupil* or schoolchild* or "school NEXT child")
(non-randomi* or norandomi* or nonRCT or non-RCT or quasi-random* or quazi-random* or quasi-experiment* or quazi-experiment* or "natural experiment" or "interrupted time series" or "controlled before and after" or "controlled before after" or "difference in difference")
#5 and #6 and #7

Table S2. Excluded systematic review records

Study ID	Full reference	Reason for exclusion
**Aceves-Martins 2016**	Aceves-Martins, M., E. Llauradó, L. Tarro, C. F. Moreno-García, T. G. Trujillo Escobar, R. Solà and M. Giralt (2016). "Effectiveness of social marketing strategies to reduce youth obesity in European school-based interventions: a systematic review and meta-analysis." Nutrition reviews 74(5): 337-351.	Ineligible study design
**Ahmad 2017**	Ahmad, S., S. Shanmugasegaram, K. Walker and S. Prince (2017). "Examining sedentary time as a risk factor for cardiometabolic diseases and their markers in South Asian adults: A systematic review." International Journal of Public Health 62(4): 503-515.	Ineligible population
**Ahmed 2021**	Ahmed, K., R. Uddin, Kolbe-Alex, T. er and A. Khan (2021). "The effectiveness of physical activity interventions in Asian children and adolescents: A systematic review." Public Health: 48-59.	Ineligible outcome
**Akcay 2024**	Akcay D, Baris N. Evaluating the effectiveness of interventions to reducing screen time in children: Meta-analysis of randomized controlled trials. Journal of Public Mental Health. 2022; 21:179-96.	Ineligible outcome
**Aldenaini 2022**	Aldenaini, N., A. Alslaity, S. Sampalli and R. Orji (2022). "Persuasive strategies and their implementations in mobile interventions for physical activity: A systematic review." International Journal of Human-Computer Interaction: No Pagination Specified.	Ineligible aim
**Allcott-Watson 2024**	Allcott-Watson H, Chater A, Troop N, Howlett N. A systematic review of interventions targeting physical activity and/or healthy eating behaviours in adolescents: Practice and training. Health Psychology Review. 2024; 18:117-40.	Ineligible outcome
**Almeida 2024**	Almeida C, Azevedo J, Fogel A, Lopes E, Vale C, Padrao P. Effectiveness of nudge interventions to promote fruit and vegetables' selection, purchase, or consumption: A systematic review. Food Quality and Preference. 2024; 116:1-14.	Ineligible outcome
**Anselma 2020**	Anselma, M., M. J. Chinapaw, D. A. Kornet-van der Aa and T. M. Altenburg (2020). "Effectiveness and promising behavior change techniques of interventions targeting energy balance related behaviors in children from lower socioeconomic environments: A systematic review." PLoS ONE Vol 15(9), 2020, ArtID e0237969.	Ineligible outcome
**Arufe-Giraldez 2022**	Arufe-Giraldez V, Sanmiguel-Rodriguez A, Ramos-Alvarez O, Navarro-Paton R. Gamification in physical education: A systematic review. Education Sciences. 2022; 12:1-20.	Ineligible outcome
**Assilian 2024**	Assilian T, Dehove H, Charreire H, Baudry J, Kesse-Guyot E, Peneau S, et al. Improving student diet and food security in higher education using participatory and co-creation approaches: A systematic review. The International Journal of Behavioral Nutrition and Physical Activity. 2024; 21.	Ineligible outcome

Study ID	Full reference	Reason for exclusion
**Audrey 2015**	Audrey, S. and H. Batista-Ferrer (2015). "Healthy urban environments for children and young people: A systematic review of intervention studies." Health & place 36: 97-117.	Ineligible study design
**Beliweiss 2023**	Bleiweiss-Sande R, Skelton K, Zaltz D, Bacardi-Gascon M, Jimenez-Cruz A, Benjamin-Neelon SE. Interventions to prevent obesity in Latinx children birth to 6 years globally: a systematic review. Public health nutrition. 2023; 26:2498-513.	Ineligible participants
**Bennett 2017**	Bennett, W. L., L. J. Cheskin, R. F. Wilson, A. Zhang, E. Tseng, O. Shogbesan, E. A. Knapp, E. A. Stuart, E. B. Bass and H. Kharrazi (2017). "Methods for Evaluating Natural Experiments in Obesity: Systematic Evidence Review."	Ineligible outcome
**Blackburn 2020**	Blackburn, N. E., J. J. Wilson, I. I. McMullan, P. Caserotti, M. Gine-Garriga, K. Wirth, L. Coll-Planas, S. B. Alias, M. Roque, M. Deidda, A. T. Kunzmann, D. Dallmeier and M. A. Tully (2020). "The effectiveness and complexity of interventions targeting sedentary behaviour across the lifespan: A systematic review and meta-analysis." The International Journal of Behavioral Nutrition and Physical Activity Vol 17 2020, ArtID 53.	Ineligible outcome
**Bleich 2013**	Bleich, S. N., J. Segal, Y. Wu, R. Wilson and Y. Wang (2013). "Systematic review of community-based childhood obesity prevention studies." Pediatrics 132(1): e201-210.	Ineligible population
**Bonvicini 2022**	Bonvicini, L., I. Pingani, F. Venturelli, N. Patrignani, M. C. Bassi, S. Broccoli, F. Ferrari, T. Gallelli, C. Panza, M. Vicentini and P. Giorgi Rossi (2022). "Effectiveness of mobile health interventions targeting parents to prevent and treat childhood Obesity: Systematic review." Preventive medicine reports 29: 101940.	Ineligible population
**Broers 2017**	Broers, V. J., C. De Breucker, S. Van den Broucke and O. Luminet (2017). "A systematic review and meta-analysis of the effectiveness of nudging to increase fruit and vegetable choice." European Journal of Public Health 27(5): 912-920.	Ineligible outcome
**Campbell 2002**	Campbell, K., E. Waters, S. O'Meara, S. Kelly and C. Summerbell (2002). "Interventions for preventing obesity in children." The Cochrane database of systematic reviews (2): CD001871.	Ineligible study design
**Carlin 2016**	Carlin, A., M. H. Murphy and A. M. Gallagher (2016). "Do Interventions to Increase Walking Work? A Systematic Review of Interventions in Children and Adolescents." Sports medicine (Auckland, N.Z.) 46(4): 515-530.	Ineligible outcome
**Carson 2016**	Carson, V., S. Hunter, N. Kuzik, C. E. Gray, V. J. Poitras, J.-P. Chaput, T. J. Saunders, P. T. Katzmarzyk, A. D. Okely, S. Connor Gorber, M. E. Kho, M. Sampson, H. Lee and M. S. Tremblay (2016). "Systematic review of sedentary behaviour and health indicators in school-aged children and youth: an update." Applied physiology, nutrition, and metabolism = Physiologie appliquee, nutrition et metabolisme 41(6): S240-265.	Ineligible study design
**Chambers 2015**	Chambers, S. A., R. Freeman, A. S. Anderson and S. MacGillivray (2015). "Reducing the volume, exposure and negative impacts of advertising for foods high in fat, sugar and salt to children: A systematic review of the evidence from statutory and self-regulatory actions and	Ineligible outcome

Study ID	Full reference	Reason for exclusion
	educational measures." Preventive Medicine: An International Journal Devoted to Practice and Theory: 32-43.	
**Chan 2022**	Chan, C. L., P. Y. Tan and Y. Y. Gong (2022). "Evaluating the impacts of school garden-based programmes on diet and nutrition-related knowledge, attitudes and practices among the school children: a systematic review." BMC public health 22(1): 1251.	Ineligible outcome
**Chaput 2020**	Chaput, J.-P., J. Willumsen, F. Bull, R. Chou, U. Ekelund, J. Firth, R. Jago, F. B. Ortega and P. T. Katzmarzyk (2020). "2020 WHO guidelines on physical activity and sedentary behaviour for children and adolescents aged 5-17 years: Summary of the evidence." The International Journal of Behavioral Nutrition and Physical Activity Vol 17 2020, ArtID 141.	Ineligible type of publication
**Choe 2022**	Choe, S., J. Sa, J. P. Chaput and D. Kim (2021). "Effectiveness of obesity interventions among South Korean children and adolescents and importance of the type of intervention component: a meta-analysis." Clinical and experimental pediatrics.	Ineligible study design
**Claudy 2021**	Claudy, M., G. Doyle, L. Marriott, N. Campbell and G. O'Malley (2021). "Are sugar-sweetened beverage taxes effective? Reviewing the evidence through a marketing systems lens." Journal of Public Policy & Marketing 40(3): 403-418.	Ineligible outcome
**Collins 2013**	Collins, C. E., T. L. Burrows, J. Bray, R. Asher, M. Young and P. J. Morgan (2013). "Effectiveness of parent-centred interventions for the prevention and treatment of childhood overweight and obesity in community settings: A systematic review." JBI Database of Systematic Reviews and Implementation Reports 11(9): 180-257.	Ineligible type of publication
**Dabravolskaj 2020**	Dabravolskaj, J., G. Montemurro, J. P. Ekwaru, X. Y. Wu, K. Storey, S. Campbell, P. J. Veugelers and A. Ohinmaa (2020). "Effectiveness of school-based health promotion interventions prioritized by stakeholders from health and education sectors: A systematic review and meta-analysis." Preventive medicine reports 19: 101138.	Ineligible study design
**Daniels 2021**	The built and social neighborhood environment and child obesity: A systematic review of longitudinal studies	Ineligible study design
**Dankiw 2020**	Dankiw, K. A., M. D. Tsiros, K. L. Baldock and S. Kumar (2020). "The impacts of unstructured nature play on health in early childhood development: A systematic review." PLoS ONE Vol 15(2), 2020, ArtID e0229006.	Ineligible outcome
**de la Hunty 2013**	de la Hunty, A., S. Gibson and M. Ashwell (2013). "Does regular breakfast cereal consumption help children and adolescents stay slimmer? A systematic review and meta-analysis." Obesity facts 6(1): 70-85.	Ineligible population
**Delgado-Noguera 2011**	Delgado-Noguera, M., S. Tort, M. J. Martinez-Zapata and X. Bonfill (2011). "Primary school interventions to promote fruit and vegetable consumption: A systematic review and meta-analysis." Preventive Medicine: An International Journal Devoted to Practice and Theory 53(1): 3-9.	Ineligible outcome

Study ID	Full reference	Reason for exclusion
**Di Nucci 2024**	Nucci A, Silano M, Cardamone E. Adherence to Mediterranean Diet and Health Outcomes in Adolescents: An Umbrella Review. Nutrition reviews. 2024.	Ineligible study design
**Eberl 2019**	Eberl, M., L. F. Tanaka, S. J. Klug and H. E. Adamek (2019). "Football as a Health Promotion Strategy." Deutsches Arzteblatt international 116(43): 721-728.	Ineligible population
**Frerichs 2015**	Frerichs, L. (2015). "Architecture and design for healthy eating in schools." Dissertation Abstracts International: Section B: The Sciences and Engineering 75(12): No Pagination Specified.	Ineligible outcome
**Fyfe-Johnson 2021**	Fyfe-Johnson, A. L., M. F. Hazlehurst, S. P. Perrins, G. N. Bratman, R. Thomas, K. A. Garrett, K. R. Hafferty, T. M. Cullaz, E. K. Marcuse and P. S. on (2021). "Nature and children's health: A systematic review." Pediatrics Vol 148(4), 2021, ArtID e2020049155.	Ineligible study design
**Gebreslassie 2020**	Gebreslassie, M., F. Sampaio, Nystr, C., R. Ssegonja and I. Feldman (2020). "Economic evaluations of public health interventions for physical activity and healthy diet: A systematic review." Preventive Medicine: An International Journal Devoted to Practice and Theory Vol 136 2020, ArtID 106100.	Ineligible study design
**Godoy-Cumillaf 2023**	Godoy-Cumillaf A, Farías-Valenzuela C, Duclos-Bastías D, Giakoni-Ramírez F, Vásquez-Gómez J, Bruneau-Chávez J, et al. Effects of physical activity interventions on anthropometric indicators and health indices in Chilean children and adolescents: A protocol for systematic review and/or meta-analysis. Medicine. 2023; 102:e33894.	Ineligible study design
**Goodyear 2021**	Goodyear, V. A., G. Wood, B. Skinner and J. L. Thompson (2021). "The effect of social media interventions on physical activity and dietary behaviours in young people and adults: A systematic review." The International Journal of Behavioral Nutrition and Physical Activity Vol 18 2021, ArtID 72.	Ineligible population
**Gori 2017**	Gori, D., F. Guaraldi, S. Cinocca, G. Moser, P. Rucci and M. P. Fantini (2017). "Effectiveness of educational and lifestyle interventions to prevent paediatric obesity: systematic review and meta-analyses of randomized and non-randomized controlled trials." Obesity science & practice 3(3): 235-248.	Ineligible population
**Gupta 2021**	Gupta, B., D. Li, P. Dong and M. C. Acri (2021). "From intention to action: A systematic literature review of provider behaviour change-focused interventions in physical health and behavioural health settings." Journal of Evaluation in Clinical Practice 27(6): 1429-1445.	Ineligible population
**Hammersley 2016**	Hammersley, M. L., R. A. Jones and A. D. Okely (2016). "Parent-Focused Childhood and Adolescent Overweight and Obesity eHealth Interventions: A Systematic Review and Meta-Analysis." Journal of medical Internet research 18(7): e203.	Ineligible study design
**Hassen 2021**	Hassen, H. Y., R. Ndejjo, G. Musinguzi, J.-P. Van Geertruyden, S. Abrams and H. Bastiaens (2021). "Effectiveness of community-based cardiovascular disease prevention interventions to improve physical activity: A systematic review and meta-regression." Preventive Medicine:	Ineligible population

Study ID	Full reference	Reason for exclusion
	An International Journal Devoted to Practice and Theory Vol 153 2021, ArtID 106797.	
**Haynes 2022**	Haynes, E., E. Augustus, C. R. Brown, C. Guell, V. Iese, L. Jia, K. Morrissey and N. Unwin (2022). "Interventions in Small Island Developing States to improve diet, with a focus on the consumption of local, nutritious foods: a systematic review." BMJ nutrition, prevention & health 5(2): 243-253.	Ineligible population
**Hersch 2014**	Hersch, D., L. Perdue, T. Ambroz and J. L. Boucher (2014). "The impact of cooking classes on food-related preferences, attitudes, and behaviors of school-aged children: A systematic review of the evidence, 2003-2014." Preventing Chronic Disease: Public Health Research, Practice, and Policy Vol 11 2014, ArtID 140267.	Ineligible study design
**Ho 2022**	Ho RS-T, Chan EK-Y, Liu KK-Y, Wong SH-S. Active video game on children and adolescents' physical activity and weight management: A network meta-analysis. Scandinavian Journal of Medicine & Science in Sports. 2022; 32:1268-86.	Ineligible study design
**Hsu 2018**	Hsu, M. S., A. Rouf and M. Allman-Farinelli (2018). "Effectiveness and behavioral mechanisms of social media interventions for positive nutrition behaviors in adolescents: A systematic review." Journal of Adolescent Health 63(5): 531-545.	Ineligible outcome
**Hu 2020**	Hu, R., L. Mbuagbaw, J.-E. Tarride, V. De Rubeis, S. Carsley and L. N. Anderson (2020). "Methodological approaches to the design and analysis of nonrandomized intervention studies for the prevention of child and adolescent obesity." Canadian journal of public health = Revue canadienne de sante publique 111(3): 358-370.	Ineligible outcome
**Hunter 2015**	Hunter, R. F., H. Christian, J. Veitch, T. Astell-Burt, J. Hipp and J. Schipperijn (2015). "The impact of interventions to promote physical activity in urban green space: A systematic review and recommendations for future research." Social Science & Medicine: 246-256.	Ineligible outcome
**Jalali 2016**	Jalali, M. S., Z. Sharafi-Avarzaman, Rahm, H. ad and A. S. Ammerman (2016). "Social influence in childhood obesity interventions: a systematic review." Obesity reviews : an official journal of the International Association for the Study of Obesity 17(9): 820-832.	Ineligible study design
**Janssen 2010**	Janssen, I. and A. G. LeBlanc (2010). "Systematic review of the health benefits of physical activity and fitness in school-aged children and youth." The International Journal of Behavioral Nutrition and Physical Activity Vol 7 2010, ArtID 40.	Ineligible study design
**Joronen 2017**	Joronen, K., A. Aikasalo and A. Suvitie (2017). "Nonphysical effects of exergames on child and adolescent well-being: A comprehensive systematic review." Scandinavian Journal of Caring Sciences 31(3): 449-461.	Ineligible outcome
**Joseph 2016**	Joseph, R. P. and J. E. Maddock (2016). "Observational park-based physical activity studies: A systematic review of the literature." Preventive Medicine: An International Journal Devoted to Practice and Theory: 257-277.	Ineligible outcome

Study ID	Full reference	Reason for exclusion
**Kelso 2020**	Kelso, A., S. Linder, A. K. Reimers, S. J. Klug, M. Alesi, L. Scifo, C. C. Borrego, D. Monteiro and Y. Demetriou (2020). "Effects of school-based interventions on motivation towards physical activity in children and adolescents: A systematic review and meta-analysis." Psychology of Sport and Exercise Vol 51 2020, ArtID 101770.	Ineligible outcome
**Kunpeuk 2020**	Kunpeuk, W., W. Spence, S. Phulkerd, R. Suphanchaimat and S. Pitayarangsarit (2020). "The impact of gardening on nutrition and physical health outcomes: A systematic review and meta-analysis." Health Promotion International 35(2): 397-408.	Ineligible population
**Lhachimi 2020**	Lhachimi, S. K., F. Pega, T. L. Heise, C. Fenton, ida, G. Gartlehner, U. Griebler, I. Sommer, M. Bombana and S. V. Katikireddi (2020). "Taxation of the fat content of foods for reducing their consumption and preventing obesity or other adverse health outcomes." The Cochrane database of systematic reviews 9: CD012415.	Ineligible outcome
**Lima do Vale 2020**	Lima do Vale, M. R., A. Farmer, G. D. Ball, R. Gokiert, K. Maximova and J. Thorlakson (2020). "Implementation of healthy eating interventions in center-based childcare: The selection, application, and reporting of theories, models, and frameworks." American Journal of Health Promotion 34(4): 402-417.	Ineligible outcome
**Lin 2021**	Lin, Y., T. McGaughey and J. P. Wilson (2021). "Effects of programs and interventions related to the social environment on childhood and adolescent obesity: A systematic search for and scoping review of natural experiments." Health & Place Vol 72 2021, ArtID 102689.	Ineligible intervention
**Marker 2018**	Marker, A. M., R. G. Steele and A. E. Noser (2018). "Physical activity and health-related quality of life in children and adolescents: A systematic review and meta-analysis." Health Psychology 37(10): 893-903.	Ineligible outcome
**Martin 2022**	Martin, A., R. Brophy, J. Clarke, C. J. Hall, R. Jago, R. Kipping, T. Reid, B. Rigby, H. Taylor, J. White and S. A. Simpson (2022). "Environmental and practice factors associated with children's device-measured physical activity and sedentary time in early childhood education and care centres: A systematic review." The International Journal of Behavioral Nutrition and Physical Activity Vol 19 2022, ArtID 84.	Ineligible outcome
**Martins Ribeiro 2023**	Martins Ribeiro S., Bartelle Basso M. B., Massignan C., S. CL. Playful educational interventions in children and adolescents' health literacy: a systematic review. Health promotion international. 2023; 38.	Ineligible aim
**Mateo-Orcajada 2024**	Mateo-Orcajada A, Vaquero-Cristobal R, Abenza-Cano L. Mobile application interventions to increase physical activity and their effect on kinanthropometrics, body composition and fitness variables in adolescent aged 12-16 years old: An umbrella review. Child: Care, Health and Development. 2024; 50:No-Specified.	Ineligible study design
**McHale 2022**	McHale, F., K. Ng, S. Taylor, E. Bengoechea, C. Norton, D. O'Shea and C. Woods (2022). "A systematic literature review of peer-led strategies for promoting physical activity levels of	Ineligible outcome

Study ID	Full reference	Reason for exclusion
	adolescents." Health Education & Behavior 49(1): 41-53.	
**Middleton 2020**	Middleton, G., R. Golley, K. Patterson, F. Le Moal and J. Coveney (2020). "What can families gain from the family meal? A mixed-papers systematic review." Appetite Vol 153 2020, ArtID 104725.	Ineligible study design
**Militello 2018**	Militello, L. K., S. Kelly, B. M. Melnyk, L. Smith and R. Petosa (2018). "A review of systematic reviews targeting the prevention and treatment of overweight and obesity in adolescent populations." Journal of Adolescent Health 63(6): 675-687.	Ineligible outcome
**Morgan 2020**	Morgan, E. H., A. Schoonees, U. Sriram, M. Faure and R. A. Seguin-Fowler (2020). "Caregiver involvement in interventions for improving children's dietary intake and physical activity behaviors." The Cochrane database of systematic reviews 1: CD012547.	Ineligible aim
**Morris 2015**	Morris, H., H. Skouteris, S. Edwards and L. Rutherford (2015). "Obesity prevention interventions in early childhood education and care settings with parental involvement: A systematic review." Early Child Development and Care 185(8): 1283-1313.	Ineligible population
**Muckelbauer 2014**	Muckelbauer, R., C. L. Barbosa, T. Mittag, K. Burkhardt, N. Mikelaishvili and J. Muller-Nordhorn (2014). "Association between water consumption and body weight outcomes in children and adolescents: a systematic review." Obesity (Silver Spring, Md.) 22(12): 2462-2475.	Ineligible outcome
**Mukamana 2016**	Mukamana, O. and M. Johri (2016). "What is known about school-based interventions for health promotion and their impact in developing countries? A scoping review of the literature." Health Education Research 31(5): 587-602.	Ineligible aim
**Nathan 2019**	Nathan, N., L. Janssen, Sutherl, R., R. K. Hodder, C. E. L. Evans, D. Booth, S. L. Yoong, K. Reilly, M. Finch and L. Wolfenden (2019). "The effectiveness of lunchbox interventions on improving the foods and beverages packed and consumed by children at centre-based care or school: a systematic review and meta-analysis." The international journal of behavioral nutrition and physical activity 16(1): 38.	Ineligible outcome
**Neshteruk 2023**	Neshteruk C, Burkart S, Flanagan EW, Melnick E, Luecking C, Kracht CL. Policy, systems, and environmental interventions addressing physical activity in early childhood education settings: A systematic review. Preventive Medicine: An International Journal Devoted to Practice and Theory. 2023; 173:1-15.	Ineligible participants
**Nguyen 2022**	Nguyen, P., L. K.-D. Le, D. Nguyen, L. Gao, D. W. Dunstan and M. Moodie (2020). "The Effectiveness of sedentary behaviour interventions on sitting time and screen time in children and adults: An umbrella review of systematic reviews." The International Journal of Behavioral Nutrition and Physical Activity Vol 17 2020, ArtID 117.	Ineligible type of publication
**Oh 2022a**	Oh, C., B. Carducci, T. Vaivada and Z. A. Bhutta (2022). "Digital interventions for universal	Ineligible outcome

Study ID	Full reference	Reason for exclusion
	health promotion in children and adolescents: A systematic review." Pediatrics 149: S1-S30.	
**Oh 2022b**	Oh, C., B. Carducci, T. Vaivada and Z. A. Bhutta (2022). "Interventions to promote physical activity and healthy digital media use in children and adolescents: A systematic review." Pediatrics 149: S1-S20.	Ineligible outcome
**Oliveira 2020**	Oliveira, C. B., R. Z. Pinto, B. T. Saraiva, W. R. Tebar, L. Delfino, r. D., M. R. Franco, C. C. Silva and D. G. Christofaro (2020). "Effects of active video games on children and adolescents: A systematic review with meta-analysis." Scandinavian Journal of Medicine & Science in Sports 30(1): 4-12.	Ineligible study design
**Olufadewa 2023**	Olufadewa, I., M. Adesina, R. Oladele, T. Olufadewa, A. Solagbade, O. Ogundele, O. Asaolu, T. Adene, O. Oladesu, E. Lawal, J. Nnatus, D. Akinrinde and E. Opone (2023). "Interventions to reduce and prevent childhood obesity in low-income and middle-income countries: a systematic review and meta-analysis." The Lancet. Global health 11: S16.	Ineligible type of publication
**Rawal 2023**	Rawal T, Muris JWM, Mishra VK, Arora M, Tandon N, van Schayck OCP. Effect of an educational intervention on diet and physical activity among school-aged adolescents in Delhi -The i-PROMISe (PROMoting health literacy in Schools) Plus Study. Dialogues in health. 2023; 2:100123.	Ineligible type of publication
**Rocliffe 2023**	Rocliffe, P., B. O'Keeffe, L. Walsh, M. Stylianou, J. Woodforde, L. Garcia-Gonzalez, W. O'Brien, T. Coppinger, I. Sherwin, P. Mannix-McNamara and C. MacDonncha (2023). "The impact of typical school provision of physical education, physical activity and sports on adolescent physical activity behaviors: A systematic literature review." Adolescent Research Review: No Pagination Specified.	Ineligible outcome
**Rodrigo-Sanjoaquin 2022**	Rodrigo-Sanjoaquin J, Corral-Abos A, Aibar Solana A, Zaragoza Casterad J, Lhuisset L, Bois JE. Effectiveness of school-based interventions targeting physical activity and sedentary time among children: A systematic review and meta-analysis of accelerometer-assessed controlled trials. Public Health. 2022; 213:147-56.	Ineligible outcome
**Rotevatn 2020**	Rotevatn, T. A., G. Melendez-Torres, C. Overgaard, K. Peven, J. Hyldgaard Nilsen, H. Boggild and A. M. B. Hostgaard (2020). "Understanding rapid infant weight gain prevention: A systematic review of quantitative and qualitative evidence." European Journal of Public Health 30(4): 703-712.	Ineligible population
**Salam 2020**	Salam, R. A., Z. A. Padhani, J. K. Das, A. Y. Shaikh, Z. Hoodbhoy, S. M. Jeelani, Z. S. Lassi and Z. A. Bhutta (2020). "Effects of Lifestyle Modification Interventions to Prevent and Manage Child and Adolescent Obesity: A Systematic Review and Meta-Analysis." Nutrients 12(8).	Ineligible study design
**Saunders 2013**	Saunders, L. E., J. M. Green, M. P. Petticrew, R. Steinbach and H. Roberts (2013). "What are the health benefits of active travel? A systematic review of trials and cohort studies." PloS one 8(8): e69912.	Ineligible study design

Study ID	Full reference	Reason for exclusion
**Sawka 2013**	Sawka, K. J., G. R. McCormack, A. Nettel-Aguirre, P. Hawe and P. K. Doyle-Baker (2013). "Friendship networks and physical activity and sedentary behavior among youth: A systematized review." The International Journal of Behavioral Nutrition and Physical Activity Vol 10 2013, ArtID 130.	Ineligible outcome
**Schipperijn 2024**	Schipperijn J, Madsen CD, Toftager M, Johansen DN, Lousen I, Amholt TT, et al. The role of playgrounds in promoting children's health-A scoping review. The International Journal of Behavioral Nutrition and Physical Activity. 2024; 21.	Ineligible outcome
**Showell 2013**	Showell, N. N., O. Fawole, J. Segal, R. F. Wilson, L. J. Cheskin, S. N. Bleich, Y. Wu, B. Lau, yn and Y. Wang (2013). "A systematic review of home-based childhood obesity prevention studies." Pediatrics 132(1): e193-e200.	Ineligible study design
**Snuggs 2019**	Snuggs, S., C. Houston-Price and K. Harvey (2019). "Healthy eating interventions delivered in the family home: A systematic review." Appetite: 114-133.	Ineligible study design
**Spruit 2016**	Spruit, A., M. Assink, E. van Vugt, C. van der Put and G. Jan Stams (2016). "The effects of physical activity interventions on psychosocial outcomes in adolescents: A meta-analytic review." Clinical Psychology Review: 56-71.	Ineligible outcome
**Staniford 2012**	Staniford, L. J., J. D. Breckon, Copel and R. J. (2012). "Treatment of childhood obesity: A systematic review." Journal of Child and Family Studies 21(4): 545-564.	Ineligible population
**Starnberg 2024**	Starnberg J, Renstrom L. Scoping review showed that obesity prevention in Nordic countries had limited effects on children from birth to 7 years of age. Acta Paediatrica. 2024; 113:912-22.	Ineligible study design (unclear which studies are non-RCT)
**Swartz 2011**	Swartz, J. J., D. Braxton and A. J. Viera (2011). "Calorie menu labeling on quick-service restaurant menus: An updated systematic review of the literature." The International Journal of Behavioral Nutrition and Physical Activity Vol 8 2011, ArtID 135.	Ineligible outcome
**Tallon 2021**	Tallon, J., R. Saavedra Dias, A. Costa, J. Leitao, A. Barros, V. Rodrigues, M. Monteiro, A. Almeida, J. Narciso and A. Silva (2021). "Impact of technology and school-based nutrition education programs on nutrition knowledge and behavior during adolescence-A systematic review." Scandinavian Journal of Educational Research 65(1): 169-180.	Ineligible outcome
**van de Kop 2019**	van de Kop, J. H., W. G. van Kernebeek, R. H. Otten, H. M. Toussaint and A. P. Verhoeff (2019). "School-based physical activity interventions in prevocational adolescents: A systematic review and meta-analyses." Journal of Adolescent Health 65(2): 185-194.	Ineligible outcome
**van Grieken 2012**	van Grieken, A., N. P. Ezendam, W. D. Paulis, J. C. van der Wouden and H. Raat (2012). "Primary prevention of overweight in children and adolescents: a meta-analysis of the effectiveness of interventions aiming to decrease sedentary behaviour." The international journal of behavioral nutrition and physical activity 9: 61.	Ineligible outcome

Study ID	Full reference	Reason for exclusion
**Venturelli 2019**	Venturelli, F., F. Ferrari, S. Broccoli, L. Bonvicini, P. Mancuso, A. Bargellini and P. Giorgi Rossi (2019). "The effect of Public Health/Pediatric Obesity interventions on socioeconomic inequalities in childhood obesity: A scoping review." Obesity Reviews 20(12): 1720-1739.	Ineligible study design
**Whitt-Glover 2009**	Whitt-Glover, M. C. and S. K. Kumanyika (2009). "Systematic review of interventions to increase physical activity and physical fitness in african-americans." American Journal of Health Promotion 23(6): S33-S56.	Ineligible outcome
**Wick 2017**	Wick, K., C. S. Leeger-Aschmann, N. D. Monn, T. Radtke, L. V. Ott, C. E. Rebholz, S. Cruz, N. Gerber, E. A. Schmutz, J. J. Puder, S. Munsch, T. H. Kakebeeke, O. G. Jenni, U. Granacher and S. Kriemler (2017). "Interventions to promote fundamental movement skills in childcare and kindergarten: A systematic review and meta-analysis." Sports Medicine 47(10): 2045-2068.	Ineligible outcome
**Wolfenden 2016**	Wolfenden, L., J. Jones, C. M. Williams, M. Finch, R. J. Wyse, Kingsl, M. , F. Tzelepis, J. Wiggers, A. Williams, a. J., K. Seward, T. Small, V. Welch, D. Booth and S. L. Yoong (2016). "Strategies to improve the implementation of healthy eating, physical activity and obesity prevention policies, practices or programmes within childcare services." The Cochrane database of systematic reviews 10: CD011779.	Ineligible population
**Wolfenden 2020**	Wolfenden L, Barnes C, Jones J, Finch M, Wyse RJ, Kingsl, et al. Strategies to improve the implementation of healthy eating, physical activity and obesity prevention policies, practices or programmes within childcare services. Cochrane Database of Systematic Reviews. 2020.	Ineligible study design

Table S3. Details of 28 included systematic reviews.

Study ID	Databases searched and date of searches	Aim of systematic review	Method of risk of bias assessment of included studies	Meta-analysis included in the systematic review	ROBIS
**Atanasova 2022**	MEDLINE, Embase, PsycInfo (via Ovid), EconLit (via EBSCO)<br><br>From inception to March 2020	To systematically review and appraise the evidence on the causal impact studies on the relationship between the built food environment (i.e. both consumer and neighbourhood food environments) on both dietary intake and obesity related anthropometric outcomes (e.g. weight, BMI).	Critical appraisal skills programme protocol (CASP) checklist for randomized control trials and cohort studies[1,2]	N	Unclear
**Azevedo 2016**	MEDLINE, Embase, Cochrane Central register of Controlled Trials (CENTRAL), Cochrane Database of Systematic Reviews, Database of Abstracts of Reviews of Effects (DARE), PsycINFO, CINAHL, ERIC, SPORTDiscus.<br><br>From 1980 to March	To summarize and compare the effect of interventions that target sedentary behaviour (e.g., TV viewing, video gaming) on BMI or BMI z-score in children (0 to 17 years old of any weight status), assessed using either a randomized or non-randomized controlled trial.	RoB1[3]	Y	Low

	2015				
**Balderas-Arteaga 2024**	Pubmed/Medline, Lilacs, Scopus, ScienceDirect and Web of Science<br><br>Date of searches not reported	To systematically review the published literature reporting a school-based curriculum intervention about nutrition and physical activity and analyze their effect.	RoB1[3]	Y	High
**Barnes 2018**	PubMed, Psycinfo, EMBASE, Ovid Medline, SCOPUS, CINAHL, Sportdiscus, Informit<br><br>From inception to to December 2015	To assess the effectiveness of lifestyle behaviour change interventions targeting mothers and their daughters on participant adiposity, physical activity, fitness and dietary outcomes.	A nine-item tool adapted from the Consolidated Standards of Reporting Trials (CONSORT) statement[4] and previously used quality criteria for methodology and reporting	N	Unclear
**Bramante 2019**	PubMed, CINAHL, PsycINFO, EconLit<br><br>From 2000 to August 2017	To identify natural experiment studies that report effects of programs, policies or built environment changes on childhood BMI outcomes	Quality Assessment Tool for Quantitative Studies of Effective Public Health Practice Project[5]	N	Unclear
**Breslin 2023**	MEDLINE, Embase, Web of Science, PsycINFO, SPORTDiscus, Scopus<br><br>From 2012 (the year of The Daily Mile 's inception) to the 30th of June 2022	To identify the published literature on The Daily Mile, evaluate their methodological quality, and summarize the findings of the available evidence for The Daily Mile. Specific the review aimed to assess the effects of participating in the daily mile on children's PA levels,	A modified version of the Downs and Black checklist[6]	N	Unclear

		physical health, mental health, wellbeing, academic performance and cognitive function.			
**Brown 2015**	ASSIA, Cochrane Controlled Trials Register, Embase, Medline and Social Sciences Citation Index<br><br>From inception to January 2014	To assess the effectiveness of diet and physical activity interventions to prevent or treat obesity in South Asian children and adults and to describe the characteristics of effective interventions	Six Item Checklist Of Quality Of Execution adapted from the Quality Assessment Tool for Quantitative Studies of Effective Public Health Practice Project[5]	Y	High
**Campbell 2001**	MEDLINE Ovid CD-ROM, Psychlit Silverplatter CD-ROM, Embase (Ovid Via Bids), Bids information service including Science Citation Index, Social Science Citation Index, CINAHL ARC Service (Winspirs online), CENTRAL/CCTR<br><br>From January 1985 to October 1999	To assess the effectiveness of interventions, other than drug or surgical interventions, designed to prevent obesity in childhood.	NR	N	High
**Errisuriz 2018**	PubMed, MedLine, and PsycINFO<br><br>Date of searches not reported	To systematically review evidence related to experimental and quasi-experimental physical education-based intervention studies;	NR	N	Low
**Feng 2017**	China National Knowledge Infrastructure (CNKI), Wanfang, Vip, PubMed,	To conduct a systematic review and meta-analysis of school-based childhood obesity	Quality Assessment Tool for Quantitative Studies of Effective Public Health Practice Project[5]	Y	Unclear

	Embase, EBSCO, Springer, the Cochrane Library<br><br>From January 1990 to December 2015	interventions in mainland China published from January 1990 to December 2015.			
**Godoy-Cumillaf 2020**	Medline (via PubMed), Embase, Scopus, Web of Science and Scielo<br><br>Date of searches not reported	To compare the effect of physical activity only with that of physical activity plus diet interventions on BMI in Latin American children and adolescents.	Quality Assessment Tool for Quantitative Studies of Effective Public Health Practice Project[5]	Y	High
**Guerrero-Magana 2024**	Medline (Ovid), EMBASE (Ovid), LILACS, PsycINFO (Ovid), SciELO, and the Cochrane Central Register of Controlled Trials (CENTRAL) (Ovid)<br><br>From 1946 to January 2023	To assess the effectiveness of interventions for the prevention of body weight gain during the festive or holiday periods in children and adults	ROBINS-I[7]	Y	Low
**Jacob 2021**	Medline, ERIC, PsychINFO, CINAHL<br><br>From 2006 to June 2020	To synthesize evidence to determine the effectiveness of health education interventions delivered in school settings to prevent overweight and obesity and/ or reduce BMI in adolescents, and determine what the key features of effective interventions are	A modified version of a quality assessment rubric, based on Centre for Reviews and Dissemination guidance[8]	Y	Unclear
**Katz 2008**	MEDLINE, HealthStar, Psych Info and Embase	To determine the effectiveness of school-	NR	Y	High

	(first search); Medline, Ovid, Cinahl and PsychInfo, Cochrane Library (second search) From 1966 to<br><br>February 2000 (first search); February 2000 to October 2004 (second search)	based strategies for obesity prevention and control using methods of systematic review and meta-analysis			
**Kornet Van der Aa 2017**	PubMed, EMBASE, PsycINFO, Cochrane Library<br><br>From inception to January 2000 to February 2016	To summarize the recent evidence on the effectiveness of obesity prevention and treatment interventions targeting adolescents from disadvantaged backgrounds. To identify potential successful intervention strategies for this target group	Quality Assessment Tool for Quantitative Studies of Effective Public Health Practice Project[5]	N	Unclear
**Mack 2017**	PubMed, Medline, ERIC, Lilacs, SciElo and PsychInfo<br><br>From 1972 to June 2016	To examine the benefits and limitations of video games in childhood obesity and prevention	Bespoke tool. Quote: "For every study, a risk of bias assessment was performed using an eight-item scale. An item was rated as positive if it was presented adequately in the respective article, whereas an item was rated as negative if it was absent or inadequately described. For the items 'drop-out rate ≤ 30%' and 'missing	N	Unclear

			data imputation', the assessment also distinguished between a negative rating if not fulfilled and a 'not reported' rating if inadequately described. The items were evaluated across studies and were not numerically summarized into a final score for single studies, in accordance with the PRISMA statement"		
**Pineda 2021**	CINAHL, Embase, Global Health, MEDLINE, SciELO, Cochrane databases<br><br>From inception to January 2020	To assess the effectiveness of school food environment interventions in the prevention of childhood obesity with a focus on adiposity and dietary intake outcomes, based on studies published up to 2020	ROBINS-I[7]	Y	High
**Podnar 2021**	MEDLINE, The Cochrane Central Register of Controlled Trials (CENTRAL), Scopus, LILACS, OpenGrey, Open Access Thesis and Dissertations, Clinical Trials and the WHO International Clinical Trials Registry	To assess what types of physical activity interventions in schools are the most effective in improving obesity-related outcomes	A modified Newcastle–Ottawa Scale for non-randomized study designs[9]; RoB1[3]	Y	High

	From January 1994 to April 2019				
**Rochira 2020**	PubMed, EMBASE, Cochrane Library<br><br>From inception to February 2019	To assess the impact of school gardening projects on school-aged (6-13 years) children's data about fruit/ vegetable intake and knowledge, science achievement, physical activity levels, blood pressure and markers in blood samples.<br>To review and meta-analyse the impact of gardening projects on school-aged (6-13 years) children's anthropometric parameters (BMI and waist circumference changes)	RoB1[3]	Y	Unclear
**Smit 2023**	Embase (Ovid), Medline (Ovid), Web of Science SCI-EXPANDED & SSCI and Cochrane CENTRAL register of Trials plus hand-searched references of included studies and the Cochrane review by Brown and colleagues.<br><br>From date of inception until the 8th of June 2021	To assess the long-term effects (≥12 months follow-up post-intervention) of primary school-based obesity prevention interventions in children on multiple indicators of overweight and obesity	ROBINS-I[7]	Y	Unclear

**Spill 2024**	MEDLINE, Ultimate, ERIC, Business Source Complete, EconLit, Agricola, Academic Search Ultimate, and CAB Abstracts databases<br><br>The search was last updated on the 29th of April 2024	To assessed longitudinal studies measuring the associations of universal free school meals in the United States with school- and student-level outcomes, including meal participation rates, attendance, dietary intake, diet quality, anthropometrics, economic impacts, disciplinary actions, food waste, stigma, and shaming	ROBINS-I[7]	N	High
**Vega-Salas 2023**	MEDLINE (via PubMed), Web of Science, Cochrane Library, Scopus and the Latin American and Caribbean Health Sciences Literature<br><br>From January 2000 to September 2021	To systematically assess the effectiveness of interventions and policies targeting the school environments for preventing/reducing overweight or obesity among school children in Latin America and the Caribbean.	ROBINS-I[7]	N	Low
**von Philipsborn 2019**	MEDLINE, Embase and CENTRAL (via Ovid); Scopus, Google Scholar, Social Science Citation Index, BiblioMap, TRoPHI; eLENA, openGrey (formerly openSIGLE), ClinicalTrials.gov, ICTRP (International Clinical Trials Registry Platform),	To assess the effects of environmental interventions on the consumption of sugar-sweetened beverages and sugar-sweetened milk, diet-related anthropometric measures and health outcomes, and on any reported unintended	RoB1[3] (modified version of): EPOC-adapted Cochrane 'Risk of bias' tool	N	Low

	LILACS, SciELO Citation Index<br><br>From inception to January 2018	consequences or adverse outcomes.			
**Waters 2014**	Cochrane Central Register of Controlled Trials (CENTRAL), MEDLINE, EMBASE, PsycINFO, CINAHL<br><br>From inception to March 2010	To update the previous review and determine the effectiveness of educational, health promotion and/ or psychological/ family/ behavioural therapy/ counselling/ management interventions which focus on diet, physical activity or lifestyle support or both and were designed or had an underlying intervention to prevent obesity/ further weight gain, in children.	RoB1[3] (modified version of): EPOC-adapted Cochrane 'Risk of bias' tool	Y	Low
**Williams 2013**	Medline, EMBASE, PsychINFO, SportDISCUS, Web of Science, Education Resource Information Centre, British Education Index, Australian Education Index, Cumulative Index to Nursing and Allied Health Library, The Cochrane Library.<br><br>From inception to June	To systematically review the evidence for the effect of diet and physical activity policies on anthropometric outcomes among children aged 4-11 years.	The Newcastle-Ottawa Scale for assessing the quality of non-randomized studies in meta-analyses[9]	N	Unclear

	2011				
**Wolfenden 2014**	Medline, EMBASE, Cochrane Central Register of Controlled Trials, Google Scholar<br><br>From inception to 2011	To assess the current evidence regarding the effectiveness of population-based whole of community interventions in preventing excessive population weight gain.	RoB1[3] (modified version of)	N	Low
**Wolfenden 2017**	Cochrane Central Register of Controlled trials (CENTRAL) via Cochrane Library; MEDLINE, MEDLINE In Process, PsycINFO and Embase via OVID; ERIC via ProQuest; CINAHL via EBSCO; SCOPUS<br><br>From inception to September 2016	To examine the effectiveness of strategies aiming to improve the implementation of school-based policies, programs or practices to address child diet, physical activity, obesity, tobacco or alcohol use.	RoB1[3] (modified version of)	N	Low
**Wolfenden 2022**	Cochrane Central Register of Controlled trials (CENTRAL) via Cochrane Library; MEDLINE, MEDLINE In Process, PsycINFO and Embase via OVID; ERIC via ProQuest; CINAHL via EBSCO; SCOPUS<br><br>From inception to April 2021	To evaluate the benefits and harms of strategies aiming to improve school implementation of interventions to address student diet, physical activity, tobacco or alcohol use, and obesity. To evaluate the benefits and harms of strategies to improve intervention implementation on measures of student diet, physical activity,	RoB1[3] (modified version of)	N	Low

		obesity, tobacco use or alcohol use; describe their cost or cost-effectiveness; and any harms of strategies on schools, school staff or students.			

Abbreviations: BMI: body mass index; CASP: Critical Appraisal Skills Programme; CONSORT: Consolidated Standards of Reporting Trials; EPOC: Effective Practice and Organisation of Care; PRISMA: Preferred Reporting Items for Systematic reviews and Meta-Analyses; RoB: risk of bias; ROBINS-I: risk of bias in non-randomized studies of intervention.

Table S4. Full references of included individual studies (identified from the 28 systematic reviews).

Study ID	Review(s)	Full reference
**Adab 2014**	Brown 2015<br>Podnar 2020	Adab P, Pallan M, Cade JE, Ekelund U, Barrett T, Daley A, et al. Preventing childhood obesity, phase II feasibility study focusing on South Asians: BEACHeS. BMJ open. 2014; 4:1-12.
**Agurto 2018**	Podnar 2020	Agurto KP, Carrasco-Alarcón V, Salazar CM. Efficacy of a High-Intensity Interval Training program in corporal variables modification on preadolescent schoolchildren of a school in the city of Temuco, Chile. Rev Esp Nutr Humana Diet. 2018;22(2):149-156.
**Almas 2013**	Brown 2015	Almas A, Islam M, Jafar TH. School-based physical activity programme in preadolescent girls (9–11 years): a feasibility trial in Karachi, Pakistan. Archives of disease in childhood. 2013; 98:515-9.
**Alvirde-García 2013**	Podnar 2020<br>Vega-Salas 2023	Alvirde-Garcia U, Rodríguez-Guerrero AJ, Henao-Morán SA, Gómez-Pérez FJ, Aguilar-Salinas CA. Resultados de un programa comunitario de intervención en el estilo de vida en niños. Salud publica de Mexico. 2013; 55:406-14.
**Aparco 2017**	Podnar 2020	Aparco JP, Bautista-Olórtegui W, Pillaca J. Impact evaluation of educational-motivational intervention “como jugando” to prevent obesity in school children of cercado de lima: Results in the first year. Rev Peru Med Exp Salud Publica. 2017;34(3):386-394.
**Azevedo 2014**	Mack 2017<br>Podnar 2020	Azevedo LB, Watson DLB, Haighton C, Adams J. The effect of dance mat exergaming systems on physical activity and health – related outcomes in secondary schools: results from a natural experiment. BMC public health. 2014; 14:951-.
**Bacardi-Gascon 2012**	Azevedo 2016<br>Podnar 2020	Bacardi-Gascon M, Perez-Morales ME, Jimenez-Cruz A. A six month randomized school intervention and an 18-month follow-up intervention to prevent childhood obesity in Mexican elementary schools. Nutr Hosp 2012; 27: 755–762.
**Balas-Nakash 2010**	Podnar 2020	Balas-Nakash M, Benítez-Arciniega AD, Perichart-Perera O, Valdés-Ramos R, Vadillo-Ortega F. The effect of exercise on cardiovascular risk markers in Mexican school-aged children: comparison between two structured group routines. Salud publica de Mexico. 2010; 52:398-405.
**Bartelink 2019**	Balderas-Arteaga 2024	Bartelink, N. H. M., van Assema, P., Kremers, S. P. J., Savelberg, H. H. C. M., Oosterhoff, M., Willeboordse, M. et al. (2019) One- and two-year effects of the healthy primary school of the future on children’s dietary and physical activity behaviours: a quasi-experimental study. Nutrients, 11, 689.
**Benjamin Neelon 2015**	Podnar 2020	Benjamin Neelon SE, Namenek Brouwer RJ, Østbye T, et al. A community-based intervention increases physical activity and reduces obesity in school-age children in North Carolina. Child Obes. 2015;11(3):297-303.
**Bhave 2016**	Podnar 2020	Bhave S, Pandit A, Yeravdekar R, et al. Effectiveness of a 5-year school-based intervention programme to reduce adiposity and improve fitness and lifestyle in Indian children; the SYM-KEM study. Arch Dis Child. 2016;101(1):33-41.
**Breslin 2012**	Azevedo 2016	Breslin G, Brennan D, Rafferty R, Gallagher AM, Hanna D. The effect of a healthy lifestyle programme on 8- to 9-year-olds from social disadvantage. Arch Dis Child 2012; 97: 618–624.
**Brustio 2019**	Breslin 2023	Brustio PR, Mulasso A, Lupo C, Massasso A, Rainoldi A, Boccia G. The Daily Mile Is Able to Improve Cardiorespiratory Fitness When Practiced Three Times a Week. International journal of environmental research and public health. 2020; 17:2095-.
**Brustio 2020**	Breslin 2023	Brustio PR, Mulasso A, Marasso D, Ruffa C, Ballatore A, Moisè P, et al. The Daily Mile: 15 Minutes Running Improves the Physical Fitness of Italian Primary School Children. International journal of environmental research and public health. 2019; 16:3921-NA.

**Bumaryoum 2015**	Podnar 2020	Bumaryoum N. Kidquest Childhood Obesity Prevention program: Analysis of Its Influence on health of Rural South Dakota 5th and 6th Grade Children [Dissertation], South Dakota State University; 2015.
**Bush 2015**	Jacob 2021	Busch V, De Leeuw JRJ, Zuithoff NPA, van Yperen T, Schrijvers AJP. A Controlled Health Promoting School Study in the Netherlands: Effects After 1 and 2 Years of Intervention. Health promotion practice. 2015; 16:592-600.
**Capogrossi 2016**	Bramante 2019	Capogrossi KL, You W. The Influence of School Nutrition Programs on the Weight of Low-Income Children: A Treatment Effect Analysis. Health economics. 2016; 26:980-1000.
**Centis 2012**	Podnar 2020	Centis E, Marzocchi R, Di Luzio R, et al. A controlled, class-based multicomponent intervention to promote healthy lifestyle and to reduce the burden of childhood obesity. Pediatr Obes. 2012;7(6):436-445.
**Chang 2009**	Feng 2017	Unable to identify full reference
**Chen 2006**	Feng 2017	Chen J. Effectiveness of Project on Taking Obesity Control as Entry Point of Health Promoting School in Xiamen, Fujian. Strait J Prev Med. 2006;(05):8±10.
**Chen 2010**	Feng 2017	Chen L, Caballero B, Mitchell DC, Loria CM, Lin P-H, Champagne CM, et al. Reducing Consumption of Sugar-Sweetened Beverages Is Associated With Reduced Blood Pressure A Prospective Study Among United States Adults. Circulation. 2010; 121:2398-406.
**Coleman 2005**	Errisuriz 2018	Coleman KJ, Tiller CL, Sanchez J, Heath EM, Sy O, Milliken GA, et al. Prevention of the Epidemic Increase in Child Risk of Overweight in Low-Income Schools: The El Paso Coordinated Approach to Child Health. Archives of pediatrics & adolescent medicine. 2005; 159:217-24.
**Cui 2015**	Feng 2017	Cui X-y, Chen T-j, Ma J. Effect of obesity intervention with socio-ecological model on anthropometric measurements of children and adolescents. Beijing da xue xue bao Yi xue ban = Journal of Peking University Health sciences. 2015; 47:400-5.
**da Silva 2013**	Podnar 2020	Da Silva LSM, Fisberg M, De Souza Pires MM, Nassar SM, Sottovia CB. The effectiveness of a physical activity and nutrition education program in the prevention of overweight in schoolchildren in Criciúma, Brazil. Eur J Clin Nutr. 2013;67(11):1200-1204.
**Davis 2011**	Rochira 2020	Davis JN, Ventura EE, Cook LT, Gyllenhammer LE, Gatto NM. LA Sprouts: A Gardening, Nutrition, and Cooking Intervention for Latino Youth Improves Diet and Reduces Obesity. Journal of the American Dietetic Association. 2011; 111:1224-30.
**de Henauw 2015**	Podnar 2020	De Henauw S, Huybrechts I, De Bourdeaudhuij I, Bammann K, Barba G, Lissner L, et al. Effects of a community-oriented obesity prevention programme on indicators of body fatness in preschool and primary school children. Main results from the IDEFICS study. Obesity reviews: an official journal of the International Association for the Study of Obesity. 2015; 16:16-29.
**de Meij 2011**	Podnar 2020	De Meij JSB, Chinapaw MJM, Van Stralen MM, Van Der Wal MF, Van Dieren L, Van Mechelen W. Effectiveness of JUMP-in, a Dutch primary schoolbased community intervention aimed at the promotion of physical activity. Br J Sports Med. 2011;45(13):1052-1057.
**Donnelly 1996**	Campbell 2001<br>Errisuriz 2018<br>Podnar 2020	Donnelly JE, Jacobsen DJ, Whatley JE, Hill JO, Swift LL, Cherrington AD, et al. Nutrition and Physical Activity Program to Attenuate Obesity and Promote Physical and Metabolic Fitness in Elementary School Children. Obesity research. 1996; 4:229-43.
**Dring 2022**	Breslin 2023	Dring KJ, Hatch LM, Williams RA, Morris JG, Sunderland C, Nevill ME, et al. Effect of 5-weeks participation in The Daily Mile on cognitive function, physical fitness, and body composition in children. Scientific reports. 2022; 12:14309-NA.

**Duan 2008**	Feng 2017	Duan J, Sun H, Lu O, Song H, Guo X, Zhang H. Impact evaluation on early prevention on adulthood diseases in some schools in Beijing. Chinese Journal of School Health. 2008;(05):404±6.
**Eather 2013**	Podnar 2020	Eather N, Morgan PJ, Lubans DR. Improving the fitness and physical activity levels of primary school children: Results of the Fit-4-Fun group randomized controlled trial. PREV MED. 2013;56(1):12-19.
**Economos 2007**	Wolfenden 2014	Economos CD, Hyatt RR, Goldberg JP, Must A, Naumova EN, Collins J, et al. A community intervention reduces BMI z-score in children: Shape Up Somerville first year results. Obesity (Silver Spring, Md). 2007; 15:1325-36.
**Economos 2013**	Azevedo 2016	Economos CD, Hyatt RR, Must A et al. Shape Up Somerville two-year results: a community-based environmental change intervention sustains weight reduction in children. Prev Med 2013; 57: 322–327.
**Erfle 2015**	Podnar 2020	Erfle SE, Gamble A. Effects of Daily Physical Education on Physical Fitness and Weight Status in Middle School Adolescents. J Sch Health. 2015;85(1):27-35.
**Ermetici 2016**	Jacob 2021<br>Pineda 2021<br>Podnar 2020	Ermetici F, Zelaschi R, Briganti S, Dozio E, Gaeta M, Ambrogi F, et al. Association between a school-based intervention and adiposity outcomes in adolescents: The Italian "EAT" project. Obesity (Silver Spring, Md). 2016; 24:687-95.
**Evans 2018**	Guerrero-Magana 2024	Evans EW, Bond DS, Pierre DF, Howie WC, Wing RR, Jelalian E. Promoting health and activity in the summer trial: Implementation and outcomes of a pilot study. Preventive medicine reports. 2018; 10:87-92.
**Eyre 2016**	Podnar 2020	Eyre ELJ, Cox VM, Birch SL, Duncan MJ. An integrated curriculum approach to increasing habitual physical activity in deprived South Asian children. Eur J Sport Sci. 2016;16(3):381-390.
**Farias 2009**	Godoy-Cumillaf 2020<br>Podnar 2020	Farias, E.S.; Paula, F.; Carvalho, W.R.G.; Gonçalves, E.M.; Baldin, A.D.; Guerra, G. Efeito da atividade física programada sobre a composiçã o corporal em escolares adolescentes. J. Pediatr. (Rio J.) 2009, 85, 28–34.
**Fernandes 2009**	Waters 2014	Fernandes MM. Evaluating the Impact of School Nutrition and Physical Activity Policies on Child Health. NA. 2009; NA:NA-NA.
**Fitzpatrick 2017**	Bramante 2019	Fitzpatrick C, Datta GD, Henderson M, Gray-Donald K, Kestens Y, Barnett TA. School food environments associated with adiposity in Canadian children. International journal of obesity (2005). 2017; 41:1005-10.
**Fotu 2011**	Wolfenden 2014	Fotu K, Millar L, Mavoa H, Kremer P, Moodie M, Snowdon W, et al. Outcome results for the Ma'alahi Youth Project, a Tongan community-based obesity prevention programme for adolescents. Obesity reviews: an official journal of the International Association for the Study of Obesity. 2011; 12:41-50.
**Fritz 2016**	Podnar 2020	Fritz J, Cöster ME, Stenevi-Lundgren S, et al. A 5-year exercise program in children improves muscle strength without affecting fracture risk. Eur J Appl Physiol. 2016;116(4):707-715.
**Froberg 2018**	Jacob 2021	Fröberg A, Jonsson L, Berg C, Lindgren E-C, Korp P, Lindwall M, et al. Effects of an Empowerment-Based Health-Promotion School Intervention on Physical Activity and Sedentary Time among Adolescents in a Multicultural Area. International journal of environmental research and public health. 2018; 15:2542-NA.
**Gallota 2016**	Podnar 2020	Gallotta MC, Iazzoni S, Emerenziani GP, et al. Effects of combined physical education and nutritional programs on schoolchildren's healthy habits. PeerJ. 2016;2016(4).
**Gao 2008**	Feng 2017	Gao Y, Griffiths SM, Chan EYY. Community-based interventions to reduce overweight and obesity in China: a systematic review of the Chinese and English literature. Journal of public health (Oxford, England). 2007; 30:436-48.

**Gao 2013**	Mack 2017<br>Smit 2023	Gao Z, Hannan PJ, Xiang P, Stodden DF, Valdez VE. Video Game–Based Exercise, Latino Children's Physical Health, and Academic Achievement. American journal of preventive medicine. 2013; 44:240-6.
**Goldsby 2016**	Bramante 2019	Goldsby TU, George B, Yeager VA, Sen B, Ferdinand AO, Sims DMT, et al. Urban Park Development and Pediatric Obesity Rates: A Quasi-Experiment Using Electronic Health Record Data. International journal of environmental research and public health. 2016; 13:411-.
**Gonzalez 2014**	Vega-Salas 2023	Gonzálvez MT, Espada JP, Orgilés M, Soto DW, Sussman S. One-Year Effects of Project EX in Spain: A Classroom-Based Smoking Prevention and Cessation Intervention Program. PloS one. 2015; 10:e0130595-NA.
**Gorely 2011**	Podnar 2020<br>Smit 2023	Gorely T, Morris JG, Musson H, Brown S, Nevill AM, Nevill ME. Physical activity and body composition outcomes of the GreatFun2Run intervention at 20 month follow-up. The international journal of behavioral nutrition and physical activity. 2011; 8:74-.
**Graf 2008**	Podnar 2020	Graf C, Koch B, Falkowski G, et al. School-based prevention: Effects on obesity and physical performance after 4 years. J Sports Sci. 2008;26(10):987-994.
**Graham 2008**	Jacob 2021	Graham DJ, Schneider M, Cooper DM. Television viewing: moderator or mediator of an adolescent physical activity intervention? American journal of health promotion: AJHP. 2008; 23:88-91.
**Gutierrez-Martínez 2018**	Vega-Salas 2023	Gutiérrez-Martínez L, Martínez RG, González S, Bolívar MA, Estupiñan OV, Sarmiento OL. Effects of a strategy for the promotion of physical activity in students from Bogotá. Revista de saude publica. 2018; 52:79-.
**Hamelink-Basteen 2008**	Podnar 2020<br>Waters 2014	Hamelink-Baksteen, HoubenF, BunC, DeWitN. Prevention and reduction of overweight in primary school children [Preventie en reductie van overgewicht bij kinderen in het basisonderwijs]. Huisarta en Wetenschap 2008;51(13):651-6.
**Harrison 2006**	Azevedo 2016<br>Podnar 2020<br>Waters 2014	Harrison M, Burns CF, McGuinness M, Heslin J, Murphy NM. Influence of a health education intervention on physical activity and screen time in primary school children: 'Switch Off– Get Active'. J Sci Med Sport 2006; 9: 388–394.
**Hatzis 2010**	Podnar 2020	Hatzis CM, Papandreou C, Kafatos AG. School health education programs in Crete: Evaluation of behavioural and health indices a decade after initiation. PREV MED. 2010;51(3-4):262-267.
**Heath 2002**	Wolfenden 2022	Heath EM, Coleman KJ. Evaluation of the Institutionalization of the Coordinated Approach to Child Health (CATCH) in a U.S./Mexico Border Community. Health education & behavior : the official publication of the Society for Public Health Education. 2002; 29:444-60.
**Heelan 2009**	Podnar 2020<br>Williams 2013	Heelan KA, Abbey BM, Donnelly JE, Mayo MS, Welk GJ. Evaluation of a walking school bus for promoting physical activity in youth. Journal of physical activity & health. 2009; 6:560-7.
**Hennessy 2014**	Bramante 2019	Hennessy E, Oh A, Agurs-Collins T, Chriqui JF, Mâsse LC, Moser RP, et al. State-Level School Competitive Food and Beverage Laws Are Associated with Children's Weight Status. The Journal of school health. 2014; 84:609-16.
**Herrick 2012**	Bramante 2019<br>Podnar 2020	Herrick HM, Thompson HR, Kinder JR, Madsen KA. Use of SPARK to promote after-school physical activity. The Journal of school health. 2012; 82:457-61.
**Hoelscher 2010**	Wolfenden 2017<br>Wolfenden 2022	Hoelscher DM, Springer AE, Ranjit N, Perry CL, Evans AE, Stigler MH, et al. Reductions in Child Obesity Among Disadvantaged School Children With Community Involvement: The Travis County CATCH Trial. Obesity (Silver Spring, Md). 2010; 18:S36-44.
**Hollar 2010**	Podnar 2020<br>Rochira 2020	Hollar D, Lombardo M, Lopez-Mitnik G, Hollar TL, Almon M, Agatston AS, et al. Effective Multi-level, Multi-sector, School-based Obesity Prevention Programming Improves Weight, Blood Pressure, and Academic Performance, Especially among Low-

		Income, Minority Children. Journal of health care for the poor and underserved. 2010; 21:93-108.
**Hu 2011**	Feng 2017	Unable to identify full reference
**Jiang 2002**	Feng 2017	Jiang J, Xia X, Wu G, Tan Z, Song X, Wang L, et al. School-based Intervention for Obese Children. Chinese Journal of Child Health Care. 2002;(06):364±7.
**Jiang 2007**	Feng 2017<br>Podnar 2020	Jiang J, Xia X, Greiner T, Wu G, Lian G, Rosenqvist U. The effects of a 3-year obesity intervention in schoolchildren in Beijing. Child: care, health and development. 2007; 33:641-6.
**Johnson 2012; Sanigorski 2008**	Waters 2014<br>Williams 2013<br>Wolfenden 2014	Johnson B, Kremer P, Swinburn B, de Silva-Sanigorski A. Multilevel analysis of the Be Active Eat Well intervention: environmental and behavioural influences on reductions in child obesity risk. International journal of obesity (2005). 2012; 36:901-7.<br>Sanigorski A, Bell AC, Kremer P, Cuttler R, Swinburn B. Reducing unhealthy weight gain in children through community capacity-building: results of a quasi-experimental intervention program, Be Active Eat Well. International journal of obesity (2005). 2008; 32:1060-7.
**Jordan 2008**	Podnar 2020<br>Williams 2013	Jordan KC, Erickson ED, Cox R, Carlson EC, Heap E, Friedrichs M, et al. Evaluation of the Gold Medal Schools Program. Journal of the American Dietetic Association. 2008; 108:1916-20.
**Jurak 2013**	Smit 2023	Jurak G, Cooper AR, Leskošek B, Kovač M. Long-Term Effects of 4-Year Longitudinal School-Based Physical Activity Intervention on the Physical Fitness of Children and Youth during 7-Year Follow-Up Assessment. Central European journal of public health. 2013; 21:190-5.
**Kain 2004**	Katz 2008<br>Podnar 2020<br>Waters 2014	Kain J, Uauy R, Albala NA, Vio F, Cerda R, Leyton B. School-based obesity prevention in Chilean primary school children: methodology and evaluation of a controlled study. International journal of obesity and related metabolic disorders: Journal of the International Association for the Study of Obesity. 2004; 28:483-93.
**Kain 2008; Kain 2009**	Godoy-Cumillaf 2020<br>Podnar 2020	Kain J, Uauy R, Leyton B, Cerda R, Olivares S, Vio F. Effectiveness of a dietary and physical activity intervention to prevent obesity in school age children. Revista medica de Chile. 2008; 136:22-30.<br>Kain J, Concha F, Salazar G, Leyton B, del Pilar Rodríguez M, Ceballos X, et al. Prevención de obesidad en preescolares y escolares de escuelas Municipales de una Comuna de Santiago de Chile: proyecto piloto 2006. Archivos latinoamericanos de nutricion. 2009; 59:139-46.
**Kargarfard 2012**	Barnes 2018	Kargarfard M, Kelishadi R, Ziaee V, Ardalan G, Halabchi F, Mazaheri R, et al. The impact of an after-school physical activity program on health-related fitness of mother/daughter pairs: CASPIAN study. Preventive medicine. 2012; 54:219-23.
**Katz 2010**	Podnar 2020	Katz DL, Cushman D, Reynolds J, et al. Putting physical activity where it fits in the school day: Preliminary results of the ABC (Activity Bursts in the Classroom) for fitness program. Prev Chronic Dis. 2010;7(4).
**Kilanowski 2015**	Guerrero-Magana 2024	Kilanowski JF, Gordon NH. Making a Difference in Migrant Summer School: Testing a Healthy Weight Intervention. Public health nursing (Boston, Mass). 2015; 32:421-9.
**Klakk 2013**	Podnar 2020	Klakk H, Chinapaw M, Heidemann M, Andersen LB, Wedderkopp N. Effect of four additional physical education lessons on body composition in children aged 8-13 years - a prospective study during two school years. BMC Pediatr. 2013;13(1).
**Knox 2012**	Podnar 2020	Knox GJ, Baker JS, Davies B, et al. Effects of a novel school-based cross-curricular physical activity intervention on cardiovascular disease risk factors in 11- to 14-year-olds: The activity knowledge circuit. Am J Health Promot. 2012;27(2):75-

		83.
**Kremer 2011**	Wolfenden 2014	Kremser W. Phases of school health promotion implementation through the lens of complexity theory: lessons learnt from an Austrian case study. Health promotion international. 2010; 26:136-47.
**Lazorick 2014; Lazorick 2015**	Kornet Van der Aa 2017<br>Jacob 2021	Lazorick S, Crawford Y, Gilbird A, Fang X, Burr V, Moore V, et al. Long-term obesity prevention and the Motivating Adolescents with Technology to CHOOSE Health™ program. Childhood obesity (Print). 2013; 10:25-33.<br>Lazorick S, Fang X, Hardison GT, Crawford Y. Improved Body Mass Index Measures Following a Middle School-Based Obesity Intervention-The MATCH Program. The Journal of school health. 2015; 85:680-7.
**Li 2004**	Feng 2017	Li H, Jiang L, Chang X, Wang W. A Mid-term Effectiveness of Project Develop Health Promotion School Taking Obesity Control as Entry Point in Shenzhen City. Chinese Journal of Health Education. 2004;(06):19±22.
**Li 2014**	Feng 2017<br>Podnar 2020	Li X-h, Lin S-t, Guo H, Huang Y, Wu L-j, Zhang Z, et al. Effectiveness of a school-based physical activity intervention on obesity in school children: a nonrandomized controlled trial. BMC public health. 2014; 14:1282-.
**Linnell 2016**	Balderas-Arteaga 2024	Linnell, J. D., Smith, M. H., Briggs, M., Brian, K. M., Scherr, R. E., Dharmar, M. et al. (2016) Evaluating the relationships among teacher characteristics, implementation factors, and student outcomes of children participating in an experiential school-based nutrition program. Pedagogy in Health Promotion, 2, 256–265.
**Lionis 1991**	Katz 2008	Lionis C, Kafatos A, Vlachonikolis J, Vakaki M, Tzortzi M, Petraki A. The effects of a health education intervention program among Cretan adolescents. Preventive medicine. 1991; 20:685-99.
**Liu 2005**	Feng 2017	Unable to identify full reference
**Liu 2007; Liu 2008**	Feng 2017<br>Podnar 2020	Liu A, Hu X, Ma G, Cui Z-H, Pan Y-P, Chang S, et al. Report on childhood obesity in China (6) evaluation of a classroom-based physical activity promotion program. Biomedical and environmental sciences : BES. 2007; 20:19-23.<br>Liu A, Hu X, Ma G, et al. Evaluation of a classroom-based physical activity promoting programme. Obes Rev. 2008;9(SUPPL. 1):130-134.
**Liu 2012**	Feng 2017	Liu J, Davidson E, Bhopal R, White M, Johnson MR, Netto G, et al. Adapting Health Promotion Interventions to Meet the Needs of Ethnic Minority Groups: Mixed-Methods Evidence Synthesis. Health technology assessment (Winchester, England). 2012; 16:1-469.
**Liu 2014**	Feng 2017	Unable to identify full reference
**Llaurado 2018**	Podnar 2020	Llauradó E, Tarro L, Moriña D, Aceves-Martins M, Giralt M, Solà R. Follow-up of a healthy lifestyle education program (the EdAl study): four years after cessation of randomized controlled trial intervention. BMC public health. 2018; 18:104-.
**Lloyd 2012**	Podnar 2020	Lloyd JJ, Wyatt K, Creanor S. Behavioural and weight status outcomes from an exploratory trial of the Healthy Lifestyles Programme (HeLP): a novel school-based obesity prevention programme. BMJ open. 2012; 2:e000390-NA.
**Localio 2024**	Spill 2024	Localio AM, Knox MA, Basu A, Lindman T, Walkinshaw LP, Jones-Smith JC. Universal Free School Meals Policy and Childhood Obesity. Pediatrics. 2024; 153:NA-NA.
**Madsen 2015**	Podnar 2020	Madsen KA, Linchey J, Gerstein DE, Ross M, Myers EF, Brown K, et al. Energy Balance 4 Kids with Play: Results from a Two-Year Cluster-Randomized Trial. Childhood obesity (Print). 2015; 11:375-83.
**Manios 1998**	Errisuriz 2018	Manios Y, Kafatos A, Mamalakis G. The effects of a health education intervention initiated at first grade over a 3 year period:

		physical activity and fitness indices. Health education research. 1998; 13:593-606.
**McManus 2008**	Podnar 2020	McManus AM, Masters RSW, Laukkanen RMT, Yu CCW, Sit CHP, Ling FCM. Using heart-rate feedback to increase physical activity in children. PREV MED. 2008;47(4):402-408.
**Meszaros 2009**	Podnar 2020	Mészáros Z, Kiss K, Szmodis MB, Zsidegh M, Mavroudes M, Mészáros J. Effects of attending elevated level school physical education in 7 to 11-year-old boys. Acta Physiol Hung. 2009;96(3):349-357.
**Millar 2011**	Jacob 2021<br>Wolfenden 2014	Millar L, Kremer P, de Silva-Sanigorski A, McCabe MP, Mavoa H, Moodie M, et al. Reduction in overweight and obesity from a 3-year community-based intervention in Australia : the It's Your Move! project. Obesity reviews : an official journal of the International Association for the Study of Obesity. 2011; 12:20-8.
**Moore 2009**	Mack 2017	Moore JB, Pawloski L, Goldberg P, Kyeung MO, Stoehr A, Baghi H. Childhood Obesity Study: A Pilot Study of the Effect of the Nutrition Education Program Color My Pyramid. The Journal of school nursing : the official publication of the National Association of School Nurses. 2009; 25:230-9.
**Morris 2013**	Podnar 2020	Morris JG, Gorely T, Sedgwick MJ, Nevill A, Nevill ME. Effect of the Great Activity Programme on healthy lifestyle behaviours in 7-11 year olds. J Sports Sci. 2013;31(12):1280-1293
**Muckelbauer 2009**	von Philipsborn 2019	Muckelbauer R, Libuda L, Clausen K, Reinehr T, Kersting M. A Simple Dietary Intervention in the School Setting Decreased Incidence of Overweight in Children. Obesity facts. 2009; 2:282-5.
**Muros 2015**	Podnar 2020	Muros JJ, Zabala M, Oliveras-López MJ, et al. Effect of physical activity, nutritional education, and consumption of extra virgin olive oil on lipid, physiological, and anthropometric Profiles in a Pediatric Population. J Phys Act Health. 2015;12(9):1245-1252.
**Neumark-Sztainer 2009**	Podnar 2020	Neumark-Sztainer D, Haines J, Robinson-O'Brien R, et al. 'Ready. Set. ACTION!' A theater-based obesity prevention program for children: A feasibility study. Health Educ Res. 2009;24(3):407-420.
**Nogueira 2017**	Podnar 2020	Nogueira RC, Weeks BK, Beck BR. One-Year Follow-up of the CAPO Kids Trial: Are Physical Benefits Maintained? Pediatric exercise science. 2017; 29:486-95.
**Pablos 2018**	Podnar 2020	Pablos A, Nebot V, Vañó-Vicent V, Ceca D, Elvira L. Effectiveness of a school-based program focusing on diet and health habits taught through physical exercise. Appl Physiol Nutr Metab. 2018;43(4):331-337.
**Perez Solis 2015**	Podnar 2020	Pérez Solís D, Díaz Martín JJ, Álvarez Caro F, Suárez Tomás I, Suárez Menéndez E, Riaño Galán I. Effectiveness of a school-based program to prevent obesity. An Pediatr. 2015;83(1):19-25.
**Plachta-Danielzik 2011**	Podnar 2020	Plachta-Danielzik S, Landsberg B, Lange D, Seiberl J, Müller MJ. Eight-year follow-up of school-based intervention on childhood overweight - The Kiel obesity prevention study. Obes Facts. 2011;4(1):35-43.
**Puma 2013**	Smit 2023	Puma J, Romaniello C, Crane LA, Scarbro S, Belansky ES, Marshall JA. Long-term Student Outcomes of the Integrated Nutrition and Physical Activity Program. Journal of nutrition education and behavior. 2013; 45:635-42.
**Ramírez-López 2005**	Vega-Salas 2023	Ramírez-López E, Grijalva-Haro MI, Valencia ME, Ponce JA, Artalejo E. Impacto de un programa de desayunos escolares en la prevalencia de obesidad y factores de riesgo cardiovascular en niños sonorenses. Salud publica de Mexico. 2005; 47:126-33.
**Rausch Herscovici 2013**	Podnar 2020<br>Vega-Salas 2023	Rausch Herscovici C, Kovalskys I, Jose De Gregorio M. Gender differences and a school-based obesity prevention program in Argentina: a randomized trial. Rev Panam Salud Publica. 2013;34:75–82.
**Reed 2008**	Podnar 2020	Reed KE, Warburton DER, Macdonald HM, Naylor P-J, McKay HA. Action Schools! BC: a school-based physical activity intervention designed to decrease cardiovascular disease risk factors in children. Preventive medicine. 2008; 46:525-31.

**Resaland 2011**	Podnar 2020	Resaland GK, Anderssen SA, Holme IM, Mamen A, Andersen LB. Effects of a 2-year school-based daily physical activity intervention on cardiovascular disease risk factors: The Sogndal school-intervention study. Scand J Med Sci Sports. 2011;21(6):e122-e131.
**Robbins 2012**	Jacob 2021<br>Podnar 2020	Robbins LB, Pfeiffer KA, Maier KS, Lo YJ, Wesolek SM. Pilot Intervention to Increase Physical Activity Among Sedentary Urban Middle School Girls: A Two-Group Pretest–Posttest Quasi-Experimental Design. The Journal of school nursing : the official publication of the National Association of School Nurses. 2012; 28:302-15.
**Robbins 2020**	Jacob 2021	Robbins LB, Ling J, Wen F. Attending After-School Physical Activity Club 2 Days a Week Attenuated an Increase in Percentage Body Fat and a Decrease in Fitness Among Adolescent Girls at Risk for Obesity. American journal of health promotion : AJHP. 2020; 34:500-4.
**Ronsley 2013**	Podnar 2020	Ronsley R, Lee AS, Kuzeljevic B, Panagiotopoulos C. Healthy buddies™ reduces body mass index z-score and waist circumference in aboriginal children living in remote coastal communities. J Sch Health. 2013;83(9):605-613.
**Rosengren 2021**	Smit 2023	Rosengren BE, Lindgren E, Jehpsson L, Dencker M, Karlsson M. Musculoskeletal Benefits from a Physical Activity Program in Primary School are Retained 4 Years after the Program is Terminated. Calcified tissue international. 2021; 109:405-14.
**Salmon 2008**	Podnar 2020	Salmon J, Ball K, Hume C, Booth ML, Crawford D. Outcomes of a group-randomized trial to prevent excess weight gain, reduce screen behaviours and promote physical activity in 10-year-old children: switch-play. International journal of obesity (2005). 2008; 32:601-12.
**Scherr 2017**	Balderas-Arteaga 2024<br>Podnar 2020	Scherr RE, Linnell JD, Dharmar M, Beccarelli LM, Bergman JJ, Briggs M, et al. A Multicomponent, School-Based Intervention, the Shaping Healthy Choices Program, Improves Nutrition-Related Outcomes. Journal of nutrition education and behavior. 2017; 49:368-79.
**Schwartz 2016**	Bramante 2019<br>von Philipsborn 2019	Schwartz AE, Leardo M, Aneja S, Elbel B. Effect of a School-Based Water Intervention on Child Body Mass Index and Obesity. JAMA pediatrics. 2016; 170:220-6.
**Shi 2004**	Feng 2017	Shi J, Liu X, Tian X, Li Y. An analysis of intervention on obese students in primary schools in Beijing.<br>Chinese Journal of Health Education. 2004;(09):14±7.
**Shofan 2011**	Podnar 2020	Shofan Y, Kedar O, Branski D, Berry E, Wilschanski M. A school-based program of physical activity may prevent obesity. Eur J Clin Nutr. 2011;65(6):768-770.
**Simonetti 1986**	Campbell 2001	Simonetti D'Arca A, Tarsitani G, Cairella M, Siani V, De<br>Philippis S, Mancinelli S, Marazzi M, Palombi L. Prevention of<br>obesity in elementary and nursery school children. Public Health<br>1986; 100: 166–171.
**Sollerhed & Ejlertsson 2008**	Errisuriz 2018<br>Podnar 2020	Sollerhed A-C, Ejlertsson G. Physical benefits of expanded physical education in primary school: findings from a 3-year intervention study in Sweden. Scandinavian journal of medicine & science in sports. 2007; 18:102-7.
**Stock 2007**	Podnar 2020	Stock S, Miranda C, Evans S, et al. Healthy buddies: A novel, peer-led health promotion program for the prevention of obesity and eating disorders in children in elementary school. Pediatrics. 2007;120(4):e1059-e1068.
**Sun 2005**	Feng 2017	Sun G, Hu Y, Yang J, Wang S, Luo H, Xu H, et al. Study on school lunch intervention among primary<br>and secondary school students in Nanjing City. The sixth Public Nutrition Branch of China Nutrition

		Association, Dalian,China 2005; Dalian2005.
**Tamir 1990**	Katz 2008	Tamir D, Feurstein A, Brunner S, Halfon S-T, Reshef A, Palti H. Primary prevention of cardiovascular diseases in childhood: changes in serum total cholesterol, high density lipoprotein, and body mass index after 2 years of intervention in Jerusalem schoolchildren age 7-9 years. Preventive medicine. 1990; 19:22-30.
**Tao 2006**	Feng 2017	Unable to identify full reference
**Taylor 2008; Taylor 2007**	Podnar 2020<br>Waters 2014<br>Wolfenden 2014	Taylor RW, McAuley KA, Barbezat W, Strong A, Williams SM, Mann J. APPLE Project: 2-y findings of a community-based obesity prevention program in primary school–age children. The American journal of clinical nutrition. 2007; 86:735-42.<br>Taylor RW, McAuley KA, Barbezat W, Farmer VL, Williams SM, Mann JI. Two-year follow-up of an obesity prevention initiative in children: The APPLE project. Am J Clin Nutr. 2008;88(5):1371-1377.
**Tian 2006**	Feng 2017	Tian B, Zhang J, Lu S, Qian L, Zhang W, Zhang J. Impact evaluation on obesity control among primary school students in 4 cities in China. Chinese Journal of School Health. 2006;(10):869±71.
**Treu 2017**	Podnar 2020	Treu JA, Doughty K, Reynolds JS, Njike VY, Katz DL. Advancing school and community engagement now for disease prevention (ASCEND): A quasi-experimental trial of school-based interventions to prevent childhood obesity. Am J Health Promot. 2017;31(2):143-152.
**van Nassau 2014**	Azevedo 2016	van Nassau F, Singh AS, Cerin E et al. The Dutch obesity intervention in teenagers (DOiT) cluster controlled implementation trial: intervention effects and mediators and moderators of adiposity and energy balance-related behaviours. Int J Behav Nutr Phys Act 2014; 11: 158.
**Vilchis-Gil 2016**	Podnar 2020	Vilchis-Gil J, Klünder-Klünder M, Duque X, Flores-Huerta S. Decreased body mass index in schoolchildren after yearlong information sessions with parents reinforced with web and mobile phone resources: Community trial. J Med Internet Res. 2016;18(6).
**von Klinggraeff 2022**	Guerrero-Magana 2024	von Klinggraeff L, Dugger R, Brazendale K, Hunt ET, Moore JB, Turner-McGrievy G, et al. Healthy Summer Learners: An explanatory mixed methods study and process evaluation. Evaluation and program planning. 2022; 92:102070-.
**Wadolawska 2019**	Jacob 2021<br>Podnar 2020	Wadolowska L, Hamułka J, Kowalkowska J, Ulewicz N, Hoffmann M, Górnicka M, et al. Changes in Sedentary and Active Lifestyle, Diet Quality and Body Composition Nine Months after an Education Program in Polish Students Aged 11–12 Years: Report from the ABC of Healthy Eating Study. Nutrients. 2019; 11:331-NA.
**Wang 2014**	Feng 2017	Wang J, Lau W-cP, Wang H-J, Ma J. Evaluation of a comprehensive intervention with a behavioural modification strategy for childhood obesity prevention: a nonrandomized cluster controlled trial. BMC public health. 2015; 15:1206-.
**Weber 2017**	Podnar 2020	Weber KS, Spörkel O, Mertens M, et al. Positive Effects of Promoting Physical Activity and Balanced Diets in a Primary School Setting with a High Proportion of Migrant School Children. Exp Clin Endocrinol Diabetes. 2017;125(8):554-562.
**Wooten 2018**	Podnar 2020	Whooten RC, Perkins ME, Gerber MW, Taveras EM. Effects of Before-School Physical Activity on Obesity Prevention and Wellness. Am J Prev Med. 2018;54(4):510-518.
**Xu 2009**	Feng 2017	Unable to identify full reference
**Yang 2017**	Podnar 2020	Yang Y, Kang B, Lee EY, et al. Effect of an obesity prevention program focused on motivating environments in childhood: A school-based prospective study. Int J Obes. 2017;41(7):1027-1034
**Zeng 2019**	Atanasova 2022	Zeng, D., Thomsen, M.R., Nayga, R.M., Bennett, J.L., 2019a. Supermarket access and childhood bodyweight: evidence from

		store openings and closings. Econ. Hum. Biol. 33, 78–88.
**Zhao 2011**	Feng 2017	Unable to identify full reference
**Zheng 2010**	Feng 2017	Zheng J, Zou S, Du W, Wang J, Tao Y. Investigation on nutrition intervention effects of pupils in Pudong New Area. Chin J Child Health Care. 2010;(03):206±9

Table S5. Overlap in primary studies (NSRI) across the 28 included systematic reviews.

Systematic review / Study ID (Primary study)	Atanasova 2022	Azevedo 2016	Balderas-Arteaga 2024	Barnes 2018	Bramante 2019	Breslin 2023	Brown 2015	Campbell 2001	Errisuriz 2018	Feng 2017	Guerrero-Magana 2024	Godoy-Cumillaf 2020	Jacob 2021	Katz 2008	Kornet Van der Aa 2017	Mack 2017	Pineda 2021	Podnar 2021	Rochira 2020	Smit 2023	Spill 2024	Vega-Salas 2023	von Philipsborn 2019	Waters 2014	Williams 2013	Wolfenden 2014	Wolfenden 2017	Wolfenden 2022
Adab 2014							x											x										
Agurto 2018																		x										
Almas 2013							x																					
Alvirde-García 2013																		x				x						
Aparco 2017																		x										
Azevedo 2014																x		x										
Bacardi-Gascon 2012		x																x										
Balas-Nakash 2010																		x										
Bartelink 2019			x																									
Bauhoff 2014					x																							
Benjamin Neelon 2015																		x										
Bhave 2016																		x										
Breslin 2012		x																										
Brustio 2019						x																						
Brustio 2020						x																						
Bumaryoum 2015																		x										
Bush 2015													x															
Capogrossi 2016					x																							
Centis 2012																		x										

**Chang 2009**									x																			
**Chen 2006**									x																			
**Chen 2010**									x																			
**Coleman 2005**								x																				
**Cui 2015**									x																			
**da Silva 2013**																	x											
**Davis 2011**																		x										
**de Henauw 2015**																	x											
**de Meij 2011**																	x											
**Donnelly 1996**							x	x									x											
**Dring 2022**					x																							
**Duan 2008**									x																			
**Eather 2013**																	x											
**Economos 2007**																									x			
**Economos 2013**		x																										
**Erfle 2015**																	x											
**Ermetici 2016**												x				x	x											
**Evans 2018**										x																		
**Eyre 2016**																	x											
**Farias 2009**											x						x											
**Fernandes 2009**																							x					
**Fitzpatrick 2017**					x																							
**Fotu 2011**																									x			
**Fritz 2016**																	x											
**Froberg 2018**												x																
**Gallota 2016**																	x											
**Gao 2008**									x																			
**Gao 2013**															x				x									
**Goldsby 2016**					x																							

**Gonzalez 2014**																					x							
**Gorely 2011**																	x		x									
**Graf 2008**																	x											
**Graham 2008**																						x						
**Gutierrez-Martínez 2018**																	x						x					
**Hamelink-Basteen 2008**		x															x						x					
**Harrison 2006**																	x											
**Hatzis 2010**																											x	
**Heath 2002**																	x							x				
**Heelan 2009**					x																							
**Hennessy 2014**					x												x											
**Herrick 2012**																										x	x	
**Hoelscher 2010**																	x	x										
**Hollar 2010**										x																		
**Hu 2011**										x																		
**Jiang 2002**										x							x											
**Jiang 2007**																							x	x	x			
**Johnson 2012; Sanigorski 2008**																	x							x				
**Jordan 2008**																			x									
**Jurak 2013**														x			x						x					
**Kain 2004**												x																
**Kain 2008; Kain 2009**				x													x											
**Kargarfard 2012**																	x											
**Katz 2010**											x																	
**Kilanowski 2015**																	x											
**Klakk 2013**																	x											
**Knox 2012**																									x			
**Kremer 2011**															x													
**Lazorick 2014; Lazorick 2015**										x			x															

**Li 2004**										x								x										
**Li 2014**			x																									
**Linnell 2016**														x														
**Lionis 1991**										x																		
**Liu 2005**										x																		
**Liu 2007; Liu 2008**										x								x										
**Liu 2012**										x																		
**Liu 2014**																		x										
**Llaurado 2018**																		x										
**Lloyd 2012**																					x							
**Localio 2024**									x									x										
**Lucertini 2013**																		x										
**Madsen 2015**									x																			
**Manios 1998**																		x										
**McMannus 2008**																		x										
**Meszaros 2009**																		x										
**Millar 2011**													x													x		
**Moore 2009**																x												
**Morris 2013**																		x										
**Muckelbauer 2009**																							x					
**Muros 2015**																		x										
**Neumark-Sztainer 2009**																		x										
**Nogueira 2017**																		x										
**Pablos 2018**																		x										
**Perez Solis 2015**																		x										
**Plachta-Danielzik 2011**																		x										
**Puma 2013**																				x								
**Ramírez-López 2005**																						x						
**Rausch Herscovici 2013**																		x				x						

**Reed 2008**																	x										
**Resaland 2011**																	x										
**Robbins 2012**													x				x										
**Robbins 2020**													x														
**Ronsley 2013**																	x										
**Rosengren 2021**																	x										
**Salmon 2008**																			x								
**Scherr 2017**																	x										
**Schwartz 2016**			x														x										
**Sharma 2018**					x																		x					
**Shi 2004**																												
**Shofan 2011**										x																		
**Simonetti 1986**																	x										
**Sollerhed & Ejlertsson 2008**								x																				
**Stock 2007**									x								x										
**Sun 2005**																	x										
**Tamir 1990**										x																		
**Tao 2006**														x														
**Taylor 2008; 2007**										x																		
**Tian 2006**																	x						x		x		
**Treu 2017**										x																		
**van Nassau 2014**																	x										
**Vilchis-Gil 2016**		x																										
**von Klinggraeff 2022**																	x										
**Wadolawska 2019**											x																	
**Wang 2014**													x				x										
**Weber 2017**										x																		
**Wooten 2018**																	x										
**Xu 2009**																	x										

**Yang 2017**										x																		
**Zeng 2019**																		x										
**Zhao 2011**	x																											
**Zheng 2010**										x																		

Table S6. ROBIS assessment of included systematic review.

Review ID	Domain 1<br>Study eligibility criteria	Domain 2<br>Identification and selection of studies	Domain 3<br>Data collection and study appraisal	Domain 4<br>Synthesis and findings	Overall risk of bias
**Atanasova 2022**	Low	Low	Unclear	Unclear	Unclear
**Azevedo 2016**	Low	Low	Low	Low	Low
**Balderas-Arteaga 2024**	Low	Low	Low	High	High
**Barnes 2018**	Low	Unclear	Low	Unclear	Unclear
**Bramante 2019**	Low	Unclear	Unclear	Unclear	Unclear
**Breslin 2023**	Unclear	Low	Low	Low	Unclear
**Brown 2015**	Low	High	Low	Low	High
**Campbell 2001**	Low	High	Low	Low	High
**Errisuriz 2018**	Unclear	Unclear	Low	Low	Unclear
**Feng 2017**	Unclear	Unclear	Low	Low	Unclear
**Godoy-Cumillaf 2020**	Low	High	Low	Unclear	High
**Guerrero-Magana 2024**	Low	Low	Low	Low	Low
**Jacob 2021**	Low	Low	Unclear	Unclear	Unclear
**Katz 2008**	Low	High	unclear	Low	High
**Kornet Van der Aa 2017**	Unclear	Low	Low	Low	Unclear
**Mack 2017**	Unclear	Unclear	Low	Low	Unclear
**Pineda 2021**	Low	Unclear	High	High	High
**Podnar 2021**	Low	Unclear	Low	High	High
**Rochira 2020**	Low	Low	Unclear	Unclear	Unclear
**Smit 2023**	Low	Unclear	Low	Low	Unclear
**Spill 2024**	Low	Unclear	Low	Low	Unclear
**Vega-Salas 2023**	Low	Low	Low	Low	Low
**von Philipsborn 2019**	Low	Low	Low	Low	Low
**Waters 2014**	Low	Low	Low	Low	Low
**Williams 2013**	Low	Unclear	Low	Low	Unclear
**Wolfenden 2014**	Low	Low	Low	Low	Low
**Wolfenden 2017**	Low	Low	Low	Low	Low
**Wolfenden 2022**	Low	Low	Low	Low	Low

Table S7. Characteristics of the 136 eligible primary studies (NRSIs) identified from the 28 systematic reviews.

Study ID	Review(s)	Age group (years)	Setting	Intervention type	Mechanisms of change	BMI outcome(s)	Direction of effect	Included in meta-analyses	Overall risk of bias or level of quality**	Overall rating ***
**Adab 2014**	Brown 2015; Podnar 2020	5-11	School	Diet and physical activity	Policy	zBMI; SMD	Favours intervention	Yes	Medium risk of bias (Brown 2015); low risk of bias (Podnar 2020)	☺😐
**Agurto 2018**	Podnar 2020	5-11	School	Physical activity	Policy	zBMI	Favours comparator	Yes	Low risk of bias	☺
**Almas 2013**	Brown 2015	5-11	School	Physical activity	Policy	SMD	Favours intervention	Yes	Medium risk of bias (Brown 2015); low risk of bias (Podnar 2020)	☺😐
**Alvirde-García 2013**	Podnar 2020; Vega-Salas 2023	5-11	School	Diet and physical activity	Policy and educational	BMI percentile; SMD	Favours intervention	Yes	High risk of bias (Podnar 2020); some concerns (Vega-salas 2023)	😐☹
**Aparco 2017**	Podnar 2020	5-11	School	Diet and physical activity	Policy and educational	zBMI	Favours intervention	Yes	High risk of bias	☹
**Azevedo 2014**	Mack 2017; Podnar 2020	5-18	School	Physical activity	Policy	BMI; SMD	Favours intervention	Yes	High risk of bias (Podnar 2020); concerns over randomization and missing data imputation (Mack 2017)	☹
**Bacardi-Gascon 2012**	Azevedo 2016; Podnar 2020	5-11	School	Diet and physical activity	Educational	SMD	Favours intervention	Yes	Unclear	?

**Balas-Nakash 2010**	Podnar 2020	5-11	School	Physical activity	Policy	SMD	Favours intervention	Yes	High risk of bias	☹
**Bartelink 2019**	Balderas-Arteaga 2024	5-11	School	Diet and physical activity	Policy and educational	zBMI	Favours intervention	Yes	High risk of bias	☹
**Benjamin Neelon 2015**	Podnar 2020	5-11	School + community	Diet and physical activity	Policy	zBMI	Favours intervention	Yes	High risk of bias	☹
**Bhave 2016**	Podnar 2020	5-18	School	Diet and physical activity	Policy	zBMI	Favours comparator	Yes	High risk of bias	☹
**Breslin 2012**	Azevedo 2016	5-11	School	Diet and physical activity	Educational	SMD	Favours comparator	Yes	High risk of bias	☹
**Brustio 2019**	Breslin 2023	5-11	School	Physical activity	Policy	BMI	NR	No	Good quality	☺
**Brustio 2020**	Breslin 2023	5-11	School	Physical activity	Policy	BMI	NR	No	Good quality	☺
**Bumaryoum 2015**	Podnar 2020	5-11	School	Diet and physical activity	Educational	SMD	Favours comparator	Yes	High risk of bias	☹
**Bush 2015**	Jacob 2021	12-18	School	Diet and physical activity	Policy and educational	BMI	Favours intervention	No	High risk of bias	☹
**Capogrossi 2016**	Bramante 2019	5-11	School	Diet	Policy	zBMI	Mixed across subgroups	Yes	Low risk of bias	☺
**Centis 2012**	Podnar 2020	5-11	School	Diet and physical activity	Policy and educational	SMD	Favours intervention	Yes	High risk of bias	☹
**Chang 2009**	Feng 2017	5-11	School	Physical activity	Policy	BMI	Favours comparator	Yes	Low quality	☹
**Chen 2006**	Feng 2017	5-11	School	Diet and physical activity	Educational	%Ob; %Ow	Favours intervention	No	Low quality	☹
**Chen 2010**	Feng 2017	5-11	School	Diet and physical	Policy and educational	BMI	Favours intervention	No	Low quality	☹

				activity						
**Coleman 2005**	Errisuriz 2018	5-11	School	Physical activity	Policy	%OW/Ob	Favours intervention	No	NR	NR
**Cui 2015**	Feng 2017	5-18	School	Diet and physical activity	Policy and educational	%Ow	Favours intervention	No	Medium quality	😐
**da Silva 2013**	Podnar 2020	5-11	School	Diet and physical activity	Policy and educational	SMD	Favours intervention	Yes	High risk of bias	☹
**Davis 2011**	Rochira 2020	5-11	School	Diet and physical activity	Policy and educational	BMI; zBMI; BMI percentile	Mixed across outcomes	Yes	Fair quality	☺
**de Henauw 2015**	Podnar 2020	5-11	School + community	Diet and physical activity	Policy and educational	zBMI	Favours intervention	Yes	High risk of bias	☹
**de Meij 2011**	Podnar 2020	5-11	School + community	Diet and physical activity	Policy and educational	SMD	Favours intervention	Yes	High risk of bias	☹
**Donnelly 1996**	Campbell 2001; Errisuriz 2018; Podnar 2020	5-11	School	Diet and physical activity	Policy and educational	BMI; SMD	Favours intervention	Yes	High risk of bias	☹
**Dring 2022**	Breslin 2023	5-11	School	Physical activity	Policy	zBMI	NR	No	Good quality	☺
**Duan 2008**	Feng 2017	5-18	School	Diet and physical activity	Policy and educational	%Ob; %Ow	Mixed across outcomes	No	Low quality	☹
**Eather 2013**	Podnar 2020	5-11	School*	Diet and physical activity	Policy and educational	SMD	NR	Yes	Unclear	?
**Economos 2007**	Wolfenden 2014	5-11	School + community	Diet and physical activity	Policy and educational	zBMI	Favours comparator	No	High risk of bias	☹
**Economos 2013**	Azevedo 2016	5-11	School + community	Diet and physical	Policy and educational	SMD	Favours intervention	Yes	High risk of bias	☹

				activity						
**Erfle 2015**	Podnar 2020	5-18	School	Physical activity	Policy	SMD	Favours intervention	Yes	High risk of bias	☹
**Ermetici 2016**	Jacob 2021; Pineda 2021; Podnar 2020	12-18	School	Diet and physical activity	Policy and educational	zBMI	Favours intervention	Yes	Low risk of bias (Podnar 2020); medium risk of bias (Jacob 2021); high risk of bias (Pineda 2021)	☺😐☹
**Evans 2018**	Guerrero-Magana 2024	5-11	Community	Diet and physical activity	Policy	zBMI	Favours intervention	Yes	Moderate risk of bias	😐
**Eyre 2016**	Podnar 2020	5-11	School	Physical activity	Policy and educational	zBMI; SMD	Favours intervention	Yes	High risk of bias	☹
**Farias 2009**	Godoy-Cumillaf 2020; Podnar 2020	12-18	School	Physical activity	Policy and educational	SMD	Favours intervention	Yes	High risk of bias (Podnar 2020); moderate risk of bias (Godoy-Cumillaf 2020)	😐☹
**Fernandes 2009**	Waters 2014	5-11	School	Diet	Educational	%Ow/Ob	Favours comparator	No	High risk of bias	☹
**Fitzpatrick 2017**	Bramante 2019	5-11	School + community	Diet	Policy	zBMI	Favours comparator	Yes	Medium risk of bias (Brown 2015); low risk of bias (Podnar 2020)	☺😐
**Fotu 2011**	Wolfenden 2014	12-18	School + community	Diet and physical activity	Policy and educational	BMI; zBMI; %Ow/Ob	Favours intervention	Yes	High risk of bias	☹
**Fritz 2016**	Podnar 2020	5-11	School	Physical activity	Policy	SMD	Mixed across subgroups	Yes	Low risk of bias	☺
**Froberg 2018**	Jacob 2021	12-18	School	Diet and physical activity	Policy and educational	BMI	Favours comparator	Yes	High risk of bias	☹
**Gallota 2016**	Podnar 2020	5-11	School	Diet and physical activity	Policy and educational	zBMI	Favours intervention	Yes	High risk of bias	☹

**Gao 2008**	Feng 2017	5-11	School	Diet and physical activity	Policy and educational	BMI	Favours intervention	Yes	Low quality	☹
**Gao 2013**	Mack 2017; Smit 2023	5-11	School	Physical activity	Policy	BMI; %Ow		Yes	Serious risk of bias (Smit 2023); concerns over randomization, primary outcome, missing data imputation and power analysis (Mack 2017)	☹
**Goldsby 2016**	Bramante 2019	5-18	Community	Physical activity	Policy	zBMI	Favours comparator	Yes	Medium risk of bias (Brown 2015); low risk of bias (Podnar 2020)	☺😐
**Gonzalez 2014**	Vega-Salas 2023	5-11	School*	Diet	Policy and educational	%Ob; %Ow	Favours comparator	No	Moderate risk of bias	😐
**Gorely 2011**	Podnar 2020; Smit 2023	5-11	School	Diet and physical activity	Policy and educational	BMI; zBMI; SMD	Mixed across outcomes	Yes	High risk of bias (Podnar 2020); serious risk of bias (Smit 2023)	☹
**Graf 2008**	Podnar 2020	5-11	School	Physical activity	Policy and educational	SMD	Favours comparator	Yes	Low risk of bias	☺
**Graham 2008**	Jacob 2021	12-18	School	Physical activity	Policy	BMI percentile	NR	No	High risk of bias	☹
**Gutierrez-Martínez 2018**	Vega-Salas 2023	5-11	School	Physical activity	Policy and educational	zBMI	Favours comparator	Yes	Low risk of bias	☺
**Hamelink-Basteen 2008**	Podnar 2020; Waters 2014	5-11	School	Diet and physical activity	Educational	SMD	Favours intervention	Yes	High risk of bias	☹
**Harrison 2006**	Azevedo 2016; Podnar 2020; Waters 2014	5-11	School	Physical activity	Policy and educational	SMD	Favours intervention	Yes	High risk of bias (Azevedo 2016; Waters 2014); low risk of bias (Podnar 2020)	☺☹

**Hatzis 2010**	Podnar 2020	5-11	School	Diet and physical activity	Policy and educational	SMD	Favours intervention	Yes	Low risk of bias	☺
**Heath 2002**	Wolfenden 2022	5-11	School	Diet and physical activity	Policy and educational	BMI		No	High risk of bias	☹
**Heelan 2009**	Podnar 2020; Williams 2013	5-11	School*	Physical activity	Policy	SMD	Favours intervention	Yes	High risk of bias (Podnar 2020); concerns with blinding and loss to follow-up (Williams 2013)	☹
**Hennessy 2014**	Bramante 2019	12-18	School	Diet	Policy	%Ow/Ob	Favours comparator	No	Medium risk of bias (Brown 2015); low risk of bias (Podnar 2020)	☺😐
**Herrick 2012**	Bramante 2019; Podnar 2020	5-11	School	Physical activity	Policy	zBMI; SMD	Favours comparator	Yes	Medium risk of bias (Bramante 2019); low risk of bias (Podnar 2020)	☺😐
**Hoelscher 2010**	Wolfenden 2017; Wolfenden 2022	5-11	School	Diet and physical activity	Policy and educational	%Ob; %Ow	Favours comparator	No	High risk of bias	☹
**Hollar 2010**	Podnar 2020; Rochira 2020	5-11	School	Diet and physical activity	Policy and educational	zBMI; BMI percentile	Mixed across outcomes	Yes	High risk of bias (Podnar 2020); fair quality (Rochira 2020)	😐☹
**Hu 2011**	Feng 2017	5-11	School	Diet and physical activity	Policy and educational	BMI	Favours intervention	Yes	Medium quality	😐
**Jiang 2002**	Feng 2017	5-11	School	Diet and physical activity	Policy and educational	% Ob	Favours intervention	No	Medium quality	😐
**Jiang 2007**	Feng 2017; Podnar 2020	5-11	School	Diet and physical	Policy and educational	SMD; %Ob	Favours intervention	Yes	High risk of bias (Podnar 2020);	☹

				activity					medium-low quality (Feng 2017)	
**Johnson 2012; Sanigorski 2008 (Be Active Eat Well)**	Waters 2014; Williams 2013; Wolfenden 2014	5-11	School + community	Diet and physical activity	Policy	BMI; zBMI; %Ow/Ob	Favours intervention	Yes	High risk of bias (Waters 2014); concerns with blinding (Williams 2013)	☹
**Jordan 2008**	Podnar 2020; Williams 2013	5-11	School	Diet and physical activity	Policy	zBMI; SMD	Favours intervention	Yes	High risk of bias (Podnar 2020); concerns with baseline measurements, outcome measurement and blinding (Williams 2013)	☹
**Jurak 2013**	Smit 2023	5-11	School	Physical activity	Policy	BMI	Favours intervention	No	Serious risk of bias	☹
**Kain 2004**	Katz 2008; Podnar 2020; Waters 2014	5-11	School*	Diet and physical activity	Policy and educational	zBMI; SMD	Favours intervention	Yes	High risk of bias (Waters 2014); low risk of bias (Podnar 2020)	☺☹
**Kain 2008; Kain 2009**	Godoy-Cumillaf 2020; Podnar 2020	5-11	School	Diet and physical activity	Policy and educational	SMD	Favours intervention	Yes	High risk of bias (Podnar 2020); moderate risk of bias (Godoy-Cumillaf 2020)	😐☹
**Kargarfard 2012**	Barnes 2018	12-18	Community	Physical activity	Policy and educational	BMI	NR	No	High concerns with uncertainty of assessor blinding, lack of power calculation and summary results, estimated	☹

									effect size and precision.	
**Katz 2010**	Podnar 2020	5-11	School + community	Diet and physical activity	Policy and educational	SMD	Favours comparator	Yes	High risk of bias	☹
**Kilanowski 2015**	Guerrero-Magana 2024	5-11	School*	Diet and physical activity	Policy and educational	BMI; BMI percentile	Favours intervention	No	Critical risk of bias	☹☹
**Klakk 2013**	Podnar 2020	5-11	School	Physical activity	Policy	SMD	Favours intervention	Yes	Low risk of bias	☺
**Knox 2012**	Podnar 2020	12-18	School	Physical activity	Policy	SMD	Favours comparator	Yes	High risk of bias	☹
**Kremer 2011**	Wolfenden 2014	12-18	School + community	Diet and physical activity	Policy	BMI; zBMI; %Ow/Ob	Favours comparator	Yes	High risk of bias	☹
**Lazorick 2014; Lazorick 2015**	Kornet Van der Aa 2017; Jacob 2021	12-18	School	Diet and physical activity	Policy and educational	BMI; zBMI	Favours intervention	Yes	Moderate quality	😐
**Li 2004**	Feng 2017	5-11	School	Diet and physical activity	Policy and educational	%Ob	Favours intervention	Yes	Low quality	☹
**Li 2014**	Feng 2017; Podnar 2020	5-11	School	Physical activity	Policy	BMI; SMD	Favours intervention	Yes	Low risk of bias (Podnar 2020); medium quality (Feng 2017)	☺😐
**Linnell 2016**	Balderas-Arteaga 2024	5-11	School*	Diet	Policy and educational	BMI percentile	Favours intervention	No	High risk of bias	☹
**Lionis 1991**	Katz 2008	12-18	School*	Diet and physical activity	Policy and educational	SMD	Favours intervention	Yes	NR	-
**Liu 2005**	Feng 2017	12-18	School	Diet and physical activity	Policy and educational	%Ob	Favours intervention	No	Medium quality	😐
**Liu 2007; Liu 2008**	Feng 2017; Podnar 2020	5-11	School	Physical activity	Policy	BMI; SMD	Mixed across subgroups	Yes	High risk of bias (Podnar 2020);	☹

									low quality (Feng 2017)	
**Liu 2012**	Feng 2017	5-11	School	Diet and physical activity	Policy and educational	%Ob	Favours intervention	No	Medium quality	😐
**Liu 2014**	Feng 2017	12-18	School	Physical activity	Policy	BMI	Favours intervention	Yes	Low quality	☹
**Llaurado 2018**	Podnar 2020	12-18	School*	Diet and physical activity	Policy and educational	zBMI	Favours comparator	Yes	High risk of bias	☹
**Lloyd 2012**	Podnar 2020	5-11	School*	Diet and physical activity	Policy and educational	SMD	Favours intervention	Yes	Unclear	?
**Localio 2024**	Spill 2024	5-18	School	Diet	Policy	%Ow/Ob	Favours intervention	No	Low risk of bias	☺
**Madsen 2015**	Podnar 2020	5-11	School + community	Diet and physical activity	Policy and educational	zBMI	Favours intervention	Yes	High risk of bias	☹
**Manios 1998**	Errisuriz 2018	5-11	School	Diet and physical activity	Policy and educational	BMI	Favours intervention	No	NR	-
**McManus 2008**	Podnar 2020	5-11	School	Physical activity	Policy and educational	SMD	Favours intervention	Yes	High risk of bias	☹
**Meszaros 2009**	Podnar 2020	5-11	School	Physical activity	Policy	SMD	Favours intervention	Yes	High risk of bias	☹
**Millar 2011**	Jacob 2021; Wolfenden 2014	12-18	School + community*	Diet and physical activity	Policy and educational	BMI; zBMI; %Ow/Ob	Favours intervention	Yes	High risk of bias	☹
**Moore 2009**	Mack 2017	5-11	School	Diet and physical activity	Policy and educational	zBMI	Favours comparator	No	Concerns over randomization, primary outcome, missing data imputation and power analysis	😐
**Morris 2013**	Podnar 2020	5-11	School	Diet and physical	Policy	SMD	Favours intervention	Yes	High risk of bias	☹

				activity						
**Muckelbauer 2009**	von Philipsborn 2019	5-11	School	Diet	Policy and educational	zBMI	NR	Yes	High risk of bias	☹
**Muros 2015**	Podnar 2020	5-11	School + community	Diet and physical activity	Policy and educational	SMD	Favours intervention	Yes	High risk of bias	☹
**Neumark-Sztainer 2009**	Podnar 2020	5-11	School*	Diet and physical activity	Policy	zBMI	Favours intervention	Yes	Low risk of bias	☺
**Nogueira 2017**	Podnar 2020	5-11	School	Physical activity	Policy	SMD	Favours comparator	Yes	High risk of bias	☹
**Pablos 2018**	Podnar 2020	5-11	School	Physical activity	Policy	SMD	Favours comparator	Yes	High risk of bias	☹
**Perez Solis 2015**	Podnar 2020	5-11	School	Diet and physical activity	Policy and educational	zBMI	Favours intervention	Yes	High risk of bias	☹
**Plachta-Danielzik 2011**	Podnar 2020	5-11	School	Diet and physical activity	Educational	SMD	Mixed across subgroups	Yes	Low risk of bias	☺
**Puma 2013**	Smit 2023	5-11	School	Diet and physical activity	Policy and educational	%Ow/Ob	Favours intervention	No	Critical risk of bias	☹☹
**Ramírez-López 2005**	Vega-Salas 2023	5-11	School	Diet	Policy and educational	BMI; zBMI	Favours comparator	Yes	Serious risk of bias	☹
**Rausch Herscovici 2013**	Podnar 2020; Vega-Salas 2023	5-11	School	Diet and physical activity	Policy and educational	BMI; zBMI	Favours intervention	Yes	High risk of bias (Podnar 2020); low risk of bias (Vegas-Salas 2023)	☺☹
**Reed 2008**	Podnar 2020	5-11	School	Physical activity	Policy	SMD	Favours comparator	Yes	Low risk of bias	☺
**Resaland 2011**	Podnar 2020	5-11	School	Physical activity	Policy	SMD	Mixed across subgroups	Yes	High risk of bias	☹
**Robbins 2012**	Jacob 2021; Podnar 2020	5-11	School	Physical activity	Policy and educational	zBMI; SMD	Favours intervention	Yes	Low risk of bias	☺

**Robbins 2020**	Jacob 2021	5-11	School*	Diet and physical activity	Policy and educational	%Ow/Ob	Favours intervention	No	Low risk of bias	☺
**Ronsley 2013**	Podnar 2020	5-11	School	Diet and physical activity	Policy and educational	zBMI	Favours intervention	Yes	High risk of bias	☹
**Rosengren 2021**	Smit 2023	5-11	School	Physical activity	Policy	BMI	Favours comparator	Yes	Serious risk of bias	☹
**Salmon 2008**	Podnar 2020	5-11	School	Physical activity	Policy and educational	SMD	Favours intervention	Yes	High risk of bias	☹
**Scherr 2017**	Balderas-Arteaga 2024; Podnar 2020	5-11	School + community*	Diet and physical activity	Policy and educational	zBMI; BMI percentile	Favours intervention	Yes	High risk of bias	☹
**Schwartz 2016**	Bramante 2019; von Philipsborn 2019	5-11	School	Diet	Policy	zBMI	Favours intervention	Yes	High risk of bias	☹
**Shi 2004**	Feng 2017	5-11	School	Diet and physical activity	Policy and educational	%Ob	Favours comparator	No	Medium quality	😐
**Shofan 2011**	Podnar 2020	5-11	School	Diet and physical activity	Policy	SMD	Favours comparator	Yes	High risk of bias	☹
**Simonetti 1986**	Campbell 2001	5-11	School	Diet	Educational	%Ow/Ob	NR	No	NR	-
**Sollerhed & Ejlertsson 2008**	Errisuriz 2018; Podnar 2020	5-11	School	Physical activity	Policy	BMI; SMD	Favours intervention	Yes	High risk of bias	☹
**Stock 2007**	Podnar 2020	5-11	School	Diet and physical activity	Policy and educational	SMD	Favours intervention	Yes	High risk of bias	☹
**Sun 2005**	Feng 2017	5-18	School	Diet	Policy	%Ob; %Ow	Favours intervention	No	Low quality	☹
**Tamir 1990**	Katz 2008	5-11	School*	Diet and physical	Policy and educational	SMD	Favours intervention	Yes	NR	-

				activity						
**Tao 2006**	Feng 2017	5-11	School	Diet and physical activity	Policy and educational	% Ob	Favours intervention	No	Low quality	☹
**Taylor 2008; Taylor 2007 (APPLE project)**	Podnar 2020; Waters 2014; Wolfenden 2014	5-11	School	Diet and physical activity	Policy and educational	zBMI; SMD; %Ow/Ob	Favours intervention	Yes	Low risk of bias (Podnar 2020); high risk of bias (Waters 2014; Wolfenden 2014)	☺☹
**Tian 2006**	Feng 2017	5-11	School	Diet and physical activity	Policy and educational	%Ob	Favours intervention	No	Low quality	☹
**Treu 2017**	Podnar 2020	5-11	School*	Diet and physical activity	Policy and educational	SMD	Favours intervention	Yes	High risk of bias	☹
**van Nassau 2014**	Azevedo 2016	12-18	School*	Diet and physical activity	Policy and educational	SMD	Favours comparator	Yes	High risk of bias	☹
**Vilchis-Gil 2016**	Podnar 2020	5-11	School	Diet and physical activity	Policy and educational	SMD	Favours intervention	Yes	Low risk of bias	☺
**von Klinggraeff 2022**	Guerrero-Magana 2024	5-11	School	Diet and physical activity	Policy and educational	zBMI	Favours intervention	Yes	Moderate risk of bias	😐
**Wadolawska 2019**	Jacob 2021; Podnar 2020	5-11	School	Diet and physical activity	Educational	zBMI	Favours intervention	Yes	Low risk of bias (Jacob 2021); high risk of bias (Podnar 2020)	☺☹
**Wang 2014**	Feng 2017	5-11	School	Physical activity	Policy	BMI	Favours comparator	No	Medium quality	😐
**Weber 2017**	Podnar 2020	5-11	School	Diet and physical activity	Policy and educational	SMD	Favours comparator	Yes	High risk of bias	☹
**Wooten 2018**	Podnar 2020	5-11	School	Diet and physical	Policy and educational	zBMI	Favours intervention	Yes	High risk of bias	☹

				activity						
**Xu 2009**	Feng 2017	5-11	School	Physical activity	Policy	%Ob	Favours intervention	No	Medium-low quality	😐
**Yang 2017**	Podnar 2020	5-18	School	Diet and physical activity	Policy and educational	zBMI	Favours intervention	Yes	High risk of bias	☹
**Zeng 2019**	Atanasova 2022	5-18	Community	Diet	Policy	BMI	Mixed across subgroups	No	Low risk of bias	☺
**Zhao 2011**	Feng 2017	5-18	School	Diet and physical activity	Educational	%Ob	Favours intervention	No	Low quality	☹
**Zheng 2010**	Feng 2017	5-11	School	Diet and physical activity	Educational	%Ob; %Ow	Favours intervention	No	Medium-low quality	😐

*Also delivered in the home.

**As reported by the authors.

***As evaluated by the reviewers.

Risk of bias or quality assessment symbol: ☺ = low risk of bias/good quality; 😐 = some concern/moderate risk of bias or medium quality; ☹ = high risk of bias or low quality; LL = critical risk of bias; ? = unclear risk of bias.

Abbreviations: %Ow: proportion of people living with overweight; %Ob: proportion of people living with obesity; %Ow/Ob: proportion of people living with overweight and or obesity; BMI: body mass index, BMIp: body mass index percentile; NR: not reported (i.e., direction of effect could not be determined or risk of bias/level of quality was not reported); NSLP: National School Lunch Program; SBP: School Breakfast Program; SMD: body mass index standardized mean difference; zBMI: age- and sex-standardized body mass index.

Table S8. Syntheses across included reviews: direction of effects of interventions on BMI outcomes by setting, type of intervention and mechanism of change.

	Effect of the intervention	Age group 5-11 years (119 studies) n studies (%)	Age group 12-18 years (28 studies) n studies (%)
**Setting**			
School	Direction: favours intervention	67 (63.8)	14 (63.6)
	Direction: favours comparator	21 (20.0)	6 (27.3)
	Direction: mixed across subgroups	5 (4.8)	0 (0.0)
	Direction: mixed across outcomes	4 (3.8)	1 (4.5)
	Direction: NR	8 (7.6)	1 (4.5)
Community	Direction: favours intervention	1 (33.3)	0 (0.0)
	Direction: favours comparator	1 (33.3)	1 (33.3)
	Direction: mixed across subgroups	1 (33.3)	1 (33.3)
	Direction: mixed across outcomes	0 (0.0)	0 (0.0)
	Direction: NR	0 (0.0)	1 (33.3)
School + community	Direction: favours intervention	8 (72.7)	2 (66.7)
	Direction: favours comparator	3 (27.3)	1 (33.3)
	Direction: mixed across subgroups	0 (0.0)	0 (0.0)
	Direction: mixed across outcomes	0 (0.0)	0 (0.0)
	Direction: NR	0 (0.0)	0 (0.0)
**Type of intervention**			
Diet	Direction: favours intervention	4 (33.3)	2 (50.0)
	Direction: favours comparator	4 (33.3)	1 (25.0)
	Direction: mixed across subgroups	2 (16.7)	1 (25.0)
	Direction: mixed across outcomes	0 (0.0)	0 (0.0)
	Direction: NR	2 (16.7)	0 (0.0)
Physical activity	Direction: favours intervention	17 (48.6)	4 (50.0)

	Direction: favours comparator	11 (31.4)	2 (25.0)
	Direction: mixed across subgroups	3 (8.6)	0 (0.0)
	Direction: mixed across outcomes	0 (0.0)	0 (0.0)
	NR	4 (11.4)	2 (25.0)
Diet and physical activity	Direction: favours intervention	55 (76.4)	10 (62.5)
	Direction: favours comparator	10 (13.9)	5 (31.3)
	Direction: mixed across subgroups	1 (1.4)	0 (0.0)
	Direction: mixed across outcomes	4 (5.6)	1 (6.3)
	Direction: NR	2 (2.8)	0 (0.0)
**Mechanism of change**			
Educational	Direction: favours intervention	6 (54.5)	1 (100.0)
	Direction: favours comparator	3 (27.3)	0 (0.0)
	Direction: mixed across subgroups	1 (9.1)	0 (0.0)
	Direction: mixed across outcomes	0 (0.0)	0 (0.0)
	Direction: NR	1 (9.1)	0 (0.0)
Policy	Direction: favours intervention	22 (51.2)	5 (41.7)
	Direction: favours comparator	12 (27.9)	5 (41.7)
	Direction: mixed across subgroups	5 (11.6)	1 (8.3)
	Direction: mixed across outcomes	0 (0.0)	0 (0.0)
	Direction: NR	4 (9.3)	1 (8.3)
Educational and policy	Direction: favours intervention	48 (73.8)	10 (66.7)
	Direction: favours comparator	10 (15.4)	3 (20.0)
	Direction: mixed across subgroups	0 (0.0)	0 (0.0)
	Direction: mixed across outcomes	4 (6.2)	1 (6.7)
	Direction: NR	3 (4.6)	1 (6.7)

Abbreviations: n: number; NR: not reported (i.e., direction of effect could not be determined).

Of the 136 included studies, 11 included participants in both the 5-11 years and 12-18 years age group and therefore results from these studies were included in both age group analyses.

Table S9. Meta analysis results from RCTs and NRSIs in participants in the 5-11 years age group.

		RCTs*	NRSIs
**Intervention**	**Outcome**	**MD (95% CI); n of studies, n of participants; τ value, heterogeneity P value**	**MD (95% CI); n of studies, n of participants; τ value, heterogeneity P value**
**Diet**	**BMI**	short-term: 0.002 (−0.10, 0.10); 5 studies, 2107 participants; τ=0, P=1<br>medium-term: −0.01 (−0.15, 0.13); 9 studies, 6815 participants; τ=0.12, P=0.9<br>long-term-term: −0.17 (−0.48, 0.13); 2 studies, 945 participants; τ=0, P=0.3	0.30 (−0.06, 0.66); 1 study, 360 participants
	**zBMI**	short-term: − 0.06 (−0.13, 0.01); 8 studies, 3695 participants; τ=0.07, P=0.1<br>medium-term: −0.04 (−0.10, 0.02); 9 studies, 7048 participants; τ=0.07, P=0.2<br>long-term-term: − 0.05 (−0.10, 0.01); 7 studies, 5285 participants; τ=0.06, P=0.1	0.03 (−0.02, 0.07); 5 studies, 6076 participants; τ =0.04, P=0.01
	**BMIp**	short-term: 1.90 (−3.44, 7.24); 3 studies, 394 participants; τ=3.24, P=0.5<br>medium-term: −0.94 (−2.65, 0.78); 3 studies, 4363 participants; τ=0.76, P=0.3<br>long-term-term: −1.49 (-4.80, 1.82); 2 studies, 776 participants; τ=2.09, P=0.4	No studies reported BMIp
	**SMD**	n/a	−0.04 (−0.13, 0.05); 1 study, 439 participants

**Physical activity**	**BMI**	short-term: −0.02 (−0.17, 0.13); 14 studies, 4069 participants; τ=0.23, P=0.8<br>medium-term: −0.12 (−0.18, −0.05); 16 studies, 21286 participants; τ=0.05, P=0.0004<br>long-term-term: −0.07 (−0.24, 0.10); 8 studies, 8302 participants; τ=0.19, P=0.4	−0.16 (−0.44, 0.13); 4 studies, 2808 participants; τ=0.22, P=0.001
	**zBMI**	short-term: −0.02 (−0.07, 0.02); 6 studies, 3580 participants; τ=0.03, P=0.27<br>medium-term: −0.05 (−0.09, −0.02); 13 studies, 20600 participants; τ=0.04, P=0.003<br>long-term-term: −0.02 (-0.09, 0.04); 6 studies, 6940 participants; τ=0.06, P=0.5	−0.19 (−0.47, 0.09); 6 studies, 1592 participants; τ=0.27, P=0.008
	**BMI p**	short-term: −0.74 (−4.1, 2.62); 1 study, 27 participants<br>medium-term: −2.26 (−4.42, −0.10); 1 study, 621 participants<br>long-term-term: -0.80 (-2.74, 1.13); 3 studies, 860 participants; τ=0.78, P=0.4	No studies reported BMIp
	**SMD**	n/a	−0.07 (−0.17, 0.04); 23 studies, 24601 participants; τ=0.18, P=0.002
**Diet and physical activity**	**BMI**	short-term: −0.11 (−0.21, −0.01); 27 studies, 16066 participants; τ=0.18, P=0.03<br>medium-term: −0.11 (−0.21, 0.004); 21 studies, 17547 participants; τ=0.2, P=0.06<br>long-term-term: 0.03 (−0.11, 0.16); 16 studies, 22098 participants; τ=0.21, P=0.7	−0.16 (−0.22, −0.09); 5 studies, 5248 participants; τ=0, P=0.8

	**zBMI**	short-term: −0.03 (−0.06, 0.003); 26 studies, 12784 participants; τ=0.05, P=0.08<br>medium-term: −0.05 (−0.073, −0.02); 24 studies, 20998 participants; τ=0.05, P=0.001<br>long-term-term: −0.02 (−0.06, 0.01); 22 studies, 23594 participants; τ=0.07, P=0.2	−0.10 (−0.16, −0.04); 24 studies, 35622 participants; τ=0.16, P<0.0001
	**BMIp**	short-term: 0.74 (−0.50, 1.97); 5 studies, 1036 participants; τ=0, P=0.2<br>medium-term: −0.64 (−1.85, 0.57); 8 studies, 3823 participants; τ=1.31, P=0.3<br>long-term-term: −0.67 (−3.05, 1.72); 5 studies, 1765 participants; τ=2.36, P=0.6	−0.07 (−0.12, −0.02); 3 studies, 6555 participants; τ=0, P=0.4
	**SMD**	n/a	−0.15 (−0.26, −0.04); 30 studies, 28491 participants; τ=0.24, P<0.0001

*Results of the RCTs are from a Cochrane review of intervention to prevent obesity in children aged 5-11 years [10].

Abbreviations: BMI: body mass index; CI: confidence interval; MD: mean difference; NRSI: non-randomized study of intervention; RCT: randomized controlled trial; SMD: body mass index standardized mean difference; zBMI: age- and sex- standardized BMI; τ: tau.

Table S10. Meta analysis results from RCTs and NRSIs in participants in the 12-18 years age group.

		RCTs*	NRSIs
**Intervention**	**Outcome**	**MD (95% CI); τ, P-value**	**MD (95% CI); τ, P-value**
**Diet**	**BMI**	short-term: −0.18 (−0.41, 0.06); 3 studies, 605 participants; τ=0, P=0.8<br>medium-term: −0.65 (−1.19, −0.11); 3 studies, 900 participants; τ=0.44, P=0.0002<br>long-term: −0.30 (−1.67, 1.07); 1 study	No studies reported BMI
	**zBMI**	short-term: −0.06 (−0.13, 0.005); 5 studies, 3154 participants; τ=0.06, P=0.001<br>medium-term: 0.02 (−0.17, 0.21); 1 study, 112 participants<br>long-term: −0.14 (−0.38, 0.1); 2 studies, 1089 participants; τ=0.14, P=0.04	No studies reported zBMI
	**BMIp**	short-term: −0.05 (−1.23, 1.13); 2 studies, 453 participants; τ=0, P=0.6<br>medium-term: −1.89 (−3.95, 0.18); 2 studies, 421 participants; τ=0, P=0.5<br>long-term: −2.53 (−7.02, 1.96); 1 study, 44 participants	No studies reported BMIp
	**SMD**	n/a	No studies reported SMD
**Physical activity**	**BMI**	short-term: −0.64 (−1.86, 0.58); 6 studies, 1780 participants; τ=1.51, P=<0.0001<br>medium-term: −0.32 (−0.53, −0.11); 3 studies, 2143 participants; τ=0.1, P=0.2<br>long-term: −0.28 (−0.51, −0.05); 1 study, 985 participants	−0.18 (−0.60, 0.25); 1 study, 85 participants

	**zBMI**	short-term: 0.02 (−0.01, 0.05); 7 studies, 4718 participants; τ=0, P=0.8<br>medium-term: 0.004 (−0.04, 0.05); 6 studies, 5335 participants; τ=0.04, P=0.08<br>long-term: −0.05 (−0.12, 0.02); 1 study, 985 participants	0.03 (0.01, 0.05); 1 study, 980 participants
	**BMIp**	medium-term: −1.09 (−2.81, 0.63); 1 study, 1020 participants	No studies reported SMD
	**SMD**	n/a	−0.12 (−0.30, 0.06); 4 studies, 15676 participants; τ=0, P=0.6
**Diet and physical activity**	**BMI**	short-term: 0.03 (−0.07, 0.13); 11 studies, 3429 participants; τ=0, P=0.58<br>medium-term: 0.01 (-0.09, 0.11); 8 studies, 5612 participants; τ=0, P=0.95<br>long-term: 0.06 (−0.04, 0.16); 6 studies, 8736 participants; τ=0.1, P=0.05	−0.01 (−0.16, 0.14); 4 studies, 6800 participants; τ=0.11, P=0.017
	**zBMI**	short-term: −0.09 (−0.20, 0.02); 3 studies, 515 participants; τ=0.1, P=0.01<br>medium-term: −0.05 (−0.10, 0.01); 6 studies, 3511 participants; τ=0.05, P=0.03<br>long-term: −0.02 (−0.05, 0.01); 7 studies, 8430 participants; τ=0.02, P=0.2	−0.06 (−0.12, 0.01); 8 studies, 10293 participants; τ=0.09, P=<0.0001
	**BMIp**	short-term: −1.69 (−3.22, −0.16); 1 study, 46 participants<br>long-term: −1.05 (−2.85, 0.75); 1 study, 1368 participants	No studies reported BMIp
	**SMD**	n/a	−0.13 (−0.61, 0.35); 2 studies, 2091 participants; τ=0.32, P=0.006

*Results of the RCTs are from a Cochrane review of intervention to prevent obesity in children aged 12-18 years [11].

Abbreviations: BMI: body mass index; BMIp: body mass index percentile; CI: confidence interval; MD: mean difference; NRSI: non-randomized study of intervention; n/a: not applicable; RCT: randomized controlled trial; SMD: body mass index standardized mean difference; τ: tau; zBMI: age- and sex-standardized body mass index

References

1 Critical Appraisal Skills Programme. CASP (Cohort Studies) Checklist. [online] Available at: https://casp-uk.net/casp-tools-checklists/.

2 Critical Appraisal Skills Programme. CASP (Randomised Controlled Trial) Checklist. [online] Available at: https://casp-uk.net/casp-tools-checklists/.

3 Higgins, J. P. *et al.* The Cochrane Collaboration's tool for assessing risk of bias in randomised trials. *BMJ* **343**, d5928 (2011). https://doi.org/10.1136/bmj.d5928

4 Schulz, K. F., Altman, D. G., Moher, D. & Group, C. CONSORT 2010 statement: updated guidelines for reporting parallel group randomized trials. *Ann Intern Med* **152**, 726-732 (2010). https://doi.org/10.7326/0003-4819-152-11-201006010-00232

5 Effective Public Health Practice Project (EPHPP): Quality Assessment Tool for Quantitative Studies. *Available at: https://www.ephpp.ca/quality-assessment-tool-for-quantitative-studies/*

6 Downs, S. H. & Black, N. The feasibility of creating a checklist for the assessment of the methodological quality both of randomised and non-randomised studies of health care interventions. *J Epidemiol Community Health* **52**, 377-384 (1998). https://doi.org/10.1136/jech.52.6.377

7 Sterne, J. A. *et al.* ROBINS-I: a tool for assessing risk of bias in non-randomised studies of interventions. *BMJ* **355**, i4919 (2016). https://doi.org/10.1136/bmj.i4919

8 Systematic Reviews; CRD's guidance for undertaking reviews in health care. *Available at: https://www.york.ac.uk/crd/guidance/* (2009).

9 Wells, G. A., Shea, B., O'Connell, D. & al. The Newcastle-Ottawa Scale (NOS) for assessing the quality of nonrandomized studies in metaanalyses. Ottawa Health Research Institute. (2012).

10 Spiga, F. *et al.* Interventions to prevent obesity in children aged 5 to 11 years old. *Cochrane Database Syst Rev* **5**, CD015328 (2024). https://doi.org/10.1002/14651858.CD015328.pub2

11 Spiga, F. *et al.* Interventions to prevent obesity in children aged 12 to 18 years old. *Cochrane Database Syst Rev* **5**, CD015330 (2024). https://doi.org/10.1002/14651858.CD015330.pub2
